# Transcriptomic and proteomic analysis of oil body associated protein dynamics in the biofuel feedstock Pennycress (*Thlaspi arvense*)

**DOI:** 10.3389/fpls.2025.1530718

**Published:** 2025-02-18

**Authors:** María Ángeles Luján, Ana Claver, Patricia Lorente, M. Victoria López, Miguel Alfonso

**Affiliations:** ^1^ Department of Plant Biology, Estación Experimental de Aula Dei (EEAD)-CSIC, Zaragoza, Spain; ^2^ Department of Soil and Water Conservation, Estación Experimental de Aula Dei (EEAD)-CSIC, Zaragoza, Spain

**Keywords:** *OBAP*, oleosin, oil body, Pennycress, seed oil, seipin, TAG

## Abstract

Pennycress (*Thlaspi arvense*) is an emerging feedstock for biofuel production because of its high seed oil content enriched in erucic acid. A combination of transcriptomic and proteomic tools was used to characterize the dynamics and relative abundance of the major oil body related proteins in the Pennycress seed. Our analysis identified 21 oleosins (OLE), 6 oil body associated proteins (OBAPs), 3 SEIPINS, 3 caleosins, 3 stereolisins and 3 lipid droplet associated proteins (LDAPs) in the Pennycress genome, showing high homology with respect to Arabidopsis or rapeseed. RNA-Seq analysis on five Pennycress seed maturation stages showed that most *OLE* and *OBAP* genes increased their expression with seed maturation, coinciding with the highest accumulation of triacylglycerol. Western-blot analysis of the OLE2 protein during seed maturation confirmed this result. However, *OLE5* and *SEIPIN1* genes showed higher expression at the early stages of seed maturation, suggesting that both proteins could be particularly involved in the initial stages of oil body formation. Proteomic analysis on oil body enriched fractions from the YELLOW and MATURE late seed maturation stages showed that all oleosin proteins were highly abundant in oil bodies. Caleosins and stereolisins were also highly abundant. Our results indicate the existence of differential expression patterns of oil body related genes during Pennycress seed maturation, suggesting different roles of these proteins for the formation and stabilization of oil bodies in the Pennycress seed.

## Introduction

Oil seed crops accumulate high amounts of oil in their seeds as a source of carbon and energy necessary upon germination. The amount of oil that can be found in the seeds varies among plant species. Soybean, which is one of the most important oil commodities, has an 18-22% of oil per seed weight (Yao et al., 2020). The model plant *Arabidopsis thaliana* seeds contains a 28% oil on a weight basis (Li et al., 2006). On the contrary, other species like *Crambe abyssinica* or *Jatropha curcas* can reach higher seed-oil contents up to 35-45% of the total seed weight, respectively (Carlsson et al., 2007; Jonas et al., 2020). This heterogeneity is particularly illustrated in Brassicaceae, with seed oil content values ranging from 17 to 54% (Oblath et al., 2016; Velasco et al., 1999) and different fatty acid compositions, with species like *Camelina sativa* in which the major fatty acid species is 18:3 (Berti et al., 2016) while other species, like *Thlaspi arvense* (Pennycress), accumulate very long chain fatty acids (VLCFAs) in their seed-oil (Claver et al., 2017; Altendorf et al., 2019). The reasons of these differences are not completely understood. Oilseed plants store this oil in the form of neutral lipids like triacylglycerol (TAG) and sterol or wax esters in the form of the so-called oil bodies (OBs) or lipid droplets (LDs) (Murphy, 2001; Huang, 2018). OBs are formed by an inner accumulation of TAG surrounded by a phospholipid (PL) monolayer into which certain specific proteins are embedded or attached (Tauchi-Sato et al., 2002; Baud et al., 2009; Siloto et al., 2006; Huang, 2018). Plant OBs are thought to be derived from the endoplasmic reticulum (ER), from regions in close vicinity with other ER subdomains where TAG-synthetizing enzymes are present (Murphy and Vance, 1999; Hsieh and Huang, 2004; Huang, 2018). Nascent TAGs may accumulate in the hydrophobic regions of the ER membrane bilayer, adopting a lenticular conformation that later results in buddying and OB formation (Murphy and Vance, 1999; Hsieh and Huang, 2004; Robenek et al., 2004, 2006; Huang, 2018). Several proteins are structural components of these OBs that can be divided into three subgroups: oleosins, caleosins and stereolisins (Huang, 2018). These proteins have been related with the formation and structural stability of the OBs, avoiding coalescence during TAG accumulation and helping lipid mobilization upon seed germination (Siloto et al., 2006; Huang, 2018). Other proteins like seipins (Cai et al., 2015) or oil body associated proteins (OBAPs) (López-Ribera et al., 2014), have also been detected associated with plant OBs in the seed. Finally, lipid droplet associated proteins (LDAPs) have been shown to be minor constituents of the LD protein coat in the seed (Kretzschmar et al., 2020) and have been more related with LD formation in leaves (Brocard et al., 2017; Pyc et al., 2021) or fruit mesocarp (Horn et al., 2013).

Oleosins are the major proteins detected in plant OBs (Tzen and Huang, 1992). They are small proteins (15-26 kDa) with three well-defined domains: two N- and C- terminal hydrophilic domains that expand the PL monolayer and a central hydrophobic hairpin that penetrates the OB into TAG (Frandsen et al., 2001; Huang and Huang, 2017; Huang, 2018). Five lineages have been identified in most plant genomes including U, SL, SH, T and M (Huang and Huang, 2015; Huang, 2018; Chen et al., 2019). Universal (U) type oleosins might be derived from the P (primitive) lineage, which is mainly present in green algae. These U type oleosins gave rise to the seed specific SH (seed high molecular weight) and SL (seed low molecular weight) lineages (Huang and Huang, 2015; Huang, 2018; Chen et al., 2019). The T (tapetum) lineage is specific of the tapetum from Brassicaeae while the M lineage is associated with fruit mesocarp (Huang and Huang, 2015; Huang, 2018; Chen et al., 2019). It has been proposed that the density of oleosins in OBs may facilitate interactions between adjacent oleosin isoforms forming dimer and multimer complexes (Huang, 2018). However, very little is known of how the different oleosins are recruited in the OBs and whether the presence of some specific oleosins helps others for their incorporation into OBs. Miquel et al. (2014) analyzed the OB dynamics in several *ole* mutant backgrounds showing that the lack of specific oleosins influenced the dynamics and distribution of OBs during seed maturation in Arabidopsis. In fact, oleosins seemed to be critic for the size and number of OBs since their loss resulted in fewer and larger OBs in Arabidopsis (Siloto et al., 2006; Miquel et al., 2014) or soybean (Schmidt and Herman, 2008). However, other authors have questioned their role in OB formation given that they are absent in other lipid accumulating tissues like fruit mesocarp (Murphy and Vance, 1999) and that mutations in *OLE* genes in Arabidopsis resulted in minor reductions of TAG levels (Miquel et al., 2014).

Seipins are proteins that promote OB biogenesis in many eukaryotes including humans, mice, drosophila or yeast (Magré et al., 2001; Tian et al., 2011; Cartwright et al., 2015). In Arabidopsis, three *SEIPIN* genes have been studied by mutational analysis, showing alterations in OB number and morphology depending on the mutated isoform, with special relevance of *SEIPIN1* (Cai et al., 2015). More recently, a LDIP (lipid droplet interacting protein) was reported to interact with ER-localized seipins and LDAPs, regulating LD formation in Arabidopsis leaves (Pyc et al., 2021). A third group of proteins related with OB biogenesis are OBAPs. Very little is known about these proteins, although it has been demonstrated that OBAP1 was necessary to maintain the structure of OBs and for seed germination in Arabidopsis (López-Ribera et al., 2014).

Question arises which is the precise role of these different OB associated proteins and whether changes in their relative abundance or in the ratios between them could be responsible for the differences in seed oil accumulation found among plant species. Thus, species with a high number of oleosins like *Brassica napus* have smaller OBs than others with reduced oleosin content like *Sesamum indicum* (Tzen et al., 1993). Moreover, similar results were obtained when two maize lines with high-oil and low-oil content were studied (Ting et al., 1996). Oleosin mutants from Arabidopsis displayed aberrant phenotypes with larger OBs (Siloto et al., 2006) further suggesting a direct relationship between OB size and oleosin content. However, this question has not been addressed in other high seed-oil accumulating species. Moreover, the relative abundance and the distribution of other OB related proteins like seipins or OBAPs in relation to oleosins and how their interaction promotes OB formation and accumulation is poorly understood.

Pennycress (*Thlaspi arvense* L.) is a winter Brassicaceae that has attracted the attention of researchers as a promising alternative oilseed feedstock for biodiesel production because of its high seed oil content and fatty acid composition. Seeds contain around 29-40% oil (w/w) depending on the varieties, which is twice the amount present in other oil commodities like soybean or sunflower, and very similar to that found in Camelina (Moser, 2012; Claver et al., 2017; Altendorf et al., 2019; López et al., 2021). In this work we have characterized at the genetic and proteomic levels the different proteins associated to OBs in Pennycress. The aim of this study was to establish to which extent not only differences in the TAG biosynthetic machinery, but also in oil storage and accumulation, are behind the high seed oil content of Pennycress. In addition, these results will contribute to increase our knowledge about the biochemical and molecular determinants of the differences in seed oil content among Brassicaceae. To that end, genes encoding proteins involved in OB formation and stabilization (Oleosins, caleosins, stereolisins, seipins, OBAPs, and LDAPs) were identified in the Pennycress genome and their genomic structure and homologies with respect to orthologs in other plant species was analyzed. Expression of these genes was monitored through RNA-Seq analysis in five different stages during Pennycress seed maturation. The results enabled us to establish a temporal correlation between gene expression and TAG accumulation in the seed. Proteomic analysis was performed on OB-enriched fractions to study the relative abundance of OB-related proteins identifying those who seemed to be critical for maintaining Pennycress OBs. Integrated transcriptomic and proteomic analysis suggests the involvement and sequential interaction between different OB-related proteins during OB formation and accumulation in the Pennycress seed.

## Materials and methods

### Plant materials

Pennycress (*Thlaspi arvense L*.) seeds from the SPRING32 germ line (Nottingham Arabidopsis Stock Centre-NASC, UK) were used in this study. Seeds were germinated in plates on wet Whatman paper without addition of any other supplement. For germination, seeds were maintained for 3 days at 4 °C and then moved to a growth chamber for additional 10-14 days. Once germinated, seeds were transferred to pots containing a 75:25 mixture of substrate (peat moss, Kekkilä White 420W: vermiculite) and grown in a bioclimatic chamber under a light intensity of 120-150 µmol m^-2^ s^-1^, with a 16h/8h light/dark photoperiod at 22 °C and a relative humidity of 45%. SPRING32 seeds required no vernalization period. For seed maturation studies, plants were monitored for the opening of flowers in the inflorescence. Then, selected flowers were tagged every day to identify their flower maturation stage awaiting the complete development of the flower. Seeds from five developmental stages corresponding to GREEN (G, 12-14 days post-anthesis, DPA), GREENYELLOW (GY, 19-21 DPA), YELLOWGREEN (YG, 26-28 DPA), YELLOW (Y, 33-35 DPA) and MATURE (M, 45-50 DPA), were collected for analysis. In all cases, seedpods were collected at 10:00 AM (two hours after of the day-cycle illumination in the chamber). Seeds separated from the pods, corresponding to these different maturation stages, were harvested, frozen in liquid nitrogen and stored at -80 °C for further analysis.

### Sequence analysis and manipulation

All sequences were obtained from *Thlaspi arvense* genome (T_arvense_v2) available at NCBI datasets (Nunn et al., 2022), Arabidopsis genome (TAIR), Arabidopsis proteins (Uniprot) and *Brassica napus* genome (NCBI). Protein alignment was performed using the MUSCLE multiple alignment tool. Phylogenetic trees were generated using the MEGA11.0 software with the neighbor-joining (NJ) method, Jones-Taylor-Thornton (JTT) model with bootstraps of 1000 replicates. Conserved motifs were analyzed using MEME (https://meme-suite.org/meme/tools/meme) with the parameters: ZOOPS (Zero or one occurrence per sequence), the maximum number of motifs was 8 and the widths of motifs was 5 to 200 residues.

### RNA isolation, cDNA synthesis and qPCR expression analysis

Total RNA was isolated from 0.1 g of *Thlaspi arvense* seeds from three independent pools from five different plants corresponding to the five maturation stages analyzed (GREEN, GREENYELLOW, YELLOWGREEN, YELLOW and MATURE) using the CTAB-LiCl extraction method of Gasic et al. (2004). RNA concentration and integrity were measured in a Nanodrop 2000 UV-Vis Spectrophotometer (Thermo Scientific). cDNAs were synthesized from 3 µg of total RNA using SuperScript III Reverse Transcriptase (Fischer) and oligo dT primer, according to the manufacturer’s instructions.

Quantitative PCR (qRT-PCR) of target genes was performed using a 7500 Real Time PCR System (Applied Biosystems), SYBR Green Master Mix (Applied Biosystems) and specific primers (**Supplementary Table 1**). The Ct values were calculated relative to *ACT2* and *EF1α* reference genes using 2^-ΔΔCt^ method (Livak and Schmittgen, 2001). Data were obtained from the analysis of at least three biological samples, with three independent technical repeats for each sample.

### RNA-Seq analysis

All procedures were carried out as detailed in (Claver et al., 2024). In short, RNA-Seq libraries were prepared and sequenced on an Illumina NovaSeq6000 at Novogene Ltd (www.novogene.uk). Ten libraries corresponding to two biological replicates of the five different seed developmental stages were constructed in this work. For each library, raw reads, clean reads, quality parameters as Q20 (%), Q30 (%) and QC (%), as well as the mapped percentage were first monitored (Claver et al., 2024). Once raw reads were cleaned, alignments were performed with HISAT2 (Mortazavi et al., 2008). Mapped regions were classified as exons, introns, or intergenic regions, and annotated with respect to the Pennycress reference genome (www.ncbi.nl,.gov/assembly/GCA_91186555.2; Nunn et al., 2022). Correlation of the gene expression levels between samples was estimated by Pearson coefficient greater than 0.92 and R^2^ greater than 0.8. The quality of the data was assessed using a Pearson correlation analysis, which demonstrated that all libraries from the biological replicates were highly related and, therefore, suitable for the gene expression analysis. Gene expression level was estimated by FPKM values (short for the expected number of Fragments Per Kilobase of transcript sequence per Millions of base pairs sequenced, Mortazavi et al., 2008).

### Oil body isolation

0.3-0.4 g of *Thlaspi arvense* seeds from the two final maturation stages (YELLOW and MATURE) were homogenized in 4 ml of buffer I (0.4 M Sucrose, 10 mM KCl, 1 mM MgCl_2_, 1 mM EDTA, 100 mM HEPES, pH 7.5, 1 mM phenylmethylsulfonyl fluoride and 3µg/ml Pefabloc) with a mortar at 4°C. Homogenates were filtered through Miracloth and centrifuged at 12,000 rpm for 20 min at 4°C. The non-floating fraction was stored as cytosolic fraction A (F_A)_. The floating fraction (OB) was removed and resuspended in 200 µl of buffer II (50 mM Tris-HCl, pH 7.5 containing 8 M urea). This OB fraction was diluted with 1.5 ml of buffer III (50 mM Tris-HCl, pH 7.5) and OBs were recovered by centrifugation at 12,000 rpm for 20 min at 4°C. This step was repeated twice. The non-floating fractions from these centrifugations were stored as cytosolic fractions B and C, respectively (F_B_, F_C_). The OB final fraction was resuspended in buffer III. Protein concentration was determined using protein assay from Bio-Rad. Ten micrograms of protein were loaded per lane on a 15% SDS-PAGE gel.

### Inmunoblot analysis of OLE2 protein

Protein extracts were obtained from 0.5 g of Pennycress seeds corresponding to the five different maturation stages from GREEN to MATURE (Claver et al., 2024) as well as from enriched oil-body fractions. The powder was dissolved in buffer A (0.1 M Tris-HCl, pH: 7.5; 20% (w/v) glycerol, 1 mM EDTA; 10 mM MgCl_2_, 14 mM β-mercaptoethanol, 100 µg/ml Pefabloc (Fluka), 1 µg/ml antipain (Sigma-Aldrich) and 1 µg/ml leupeptin, (Sigma-Aldrich) and filtered with Miracloth paper (Calbiochem). The protein content of the different fractions was estimated using the BioRad protein assay reagent (BioRad). Except when specifically mentioned, total protein of 10 µg was loaded per lane. Western blot procedures were performed as described in Román et al. (2015) using OLE2 antibodies from AGRISERA at 1:1000 dilution.

### Proteomic identification by LC-TIMS-MS/MS

OB-enriched fractions from YELLOW and MATURE seed maturation stages were isolated and analyzed at the Proteomics Facility, Research Support Central Service, University of Cordoba (Spain). Protein extracts were cleaned-up in 1D SDS-PAGE at 10% of polyacrylamide. Samples were loaded in stacking gel at 4% and 100 V was applied until the electrophoresis front reached the resolving gel. Proteins were separated 1 cm in the resolving gel, the electrophoresis was finished, and the gel was stained with Coomassie Blue. Protein bands were diced and kept in water until digestion. For protein digestion, protein bands were first distained in 200 mM ammonium bicarbonate (AB)/50% acetonitrile for 15 min and then for 5 min in 100% acetonitrile. Protein was reduced by addition of 20 mM dithiothreitol in 25 mM AB and incubated for 20 min at 55°C. The mixture was cooled to room temperature, followed by alkylation of free thiols by addition of 40 mM iodoacetamide in 25 mM AB in the dark for 20 min. Then, protein bands were washed twice in 25 mM AB. Proteolytic digestion was performed by addition of Trypsin (Promega, Madison, WI), 12.5 ng/µl of enzyme in 25 mM AB and incubated at 37 °C overnight. Protein digestion was stopped by addition of trifluoroacetic acid at 1% final concentration. Samples were desalted with C18 ZipTip columns, eluted with 10 µl of 0.1% formic acid in 70% acetonitrile and resuspended in 10 µl of 0.1% FA.

LC-TIMS-MS/MS was carried out using a nanoElute nanoflow ultrahigh-pressure LC system (Bruker Daltonics, Bremen, Germany) coupled to a timsTOF Pro2 mass spectrometer, equipped with a CaptiveSpray nanoelectrospray ion source (Bruker Daltonics). For most analyzes, 200 ng of peptide digest was loaded onto an Aurora C18 capillary column (25 cm length, 75 μm ID, 1.6 μm particle size, Ion Opticks). Peptides were separated at 30°C using a 20-min gradient at a flow rate of 300 nL/min (mobile phase A (MPA): 0.1% FA; mobile phase B (MPB): 0.1% FA in acetonitrile). A step gradient from 0 to 30% MPB was applied over 24 min, followed by a 30 to 90% MPB step for 1 min, and finished with a 90% MPB wash for an additional 5 min, with a total run time of 30 min per analysis. The timsTOF Pro 2 was run in DIA-PASEF mode with isolation windows of 25 Da in a mass range of 450-950 Da without mass overlapping. Ion mobility resolution was set to 0.85–1.30 V s/cm2 over a ramp time of 100 ms. The collision energy was increased stepwise as a function of the ion mobility ramp, from 27 to 45 eV. A polygonal filter was applied on the m/z space and ion mobility to exclude low m/z, mainly single-charged ions from the selection of PASEF precursors. The raw data were analyzed in PEAKS Studio ProX (Bioinformatics Solution Corp). The reference library was acquired from NCBI_Genome_Protein_sequences. The raw data files were analyzed with parent mass error tolerance set to 15 ppm and a fragment mass error tolerance of 0.05 Da. To account for post-translational modifications and chemical labelling, the following settings were used: Carbamidomethylation of cysteine residues was set as fixed modification, methionine oxidation and Acetylation (Protein N-term) were set as variable modifications. Protein unique peptides were set to larger than 1 and a high confidence score of 1% FDR was applied to indicate an accurately identified protein. Protein intensities were determined by the intensity based absolute quantification (iBAQ) (Kretzschmar et al., 2020). iBAQ values were the calculated as per mile of all intensities for each sample to obtain relative iBAQ (riBAQ) values. When comparing proteins from both seed maturation stages, log_10_iBAQ was used with proteins with *P* value < 0.1.

### Statistical analysis

Data are expressed as means ± SD, with at least three replicates in each experimental group. The statistical comparisons among the different developmental stages during seed maturation of Pennycress were made using one-way analysis of variance (ANOVA) and means were compared with the Duncan’s multiple range test (*P* < 0.05). When data showed non-normality, log or reciprocal transformations were made and ANOVA conducted with the transformed data.

## Results

### Genome-wide identification of oleosin (*TaOLE*) family genes in the Pennycress genome

Genes encoding proteins related with oil body formation or stabilization were searched in the *Thlaspi arvense* genome available at Phytozome (v1). In the case of genes encoding oleosins, BLAST searches using different oleosin domains (Pfam 01277, PTHR33203, Interpro IPR00136) were performed, and the identified sequences were used for homologies in the *Thlaspi arvense* genome (T_arvense_v2) available at NCBI datasets (Nunn et al., 2022) using BLAST analysis. **Table 1** shows the genes identified through this analysis, indicating gene name, chromosome location, corresponding Arabidopsis ortholog as well as gene and predicted protein length. Oleosin nomenclature is still confusing, without any unified assignation for *OLE* genes and proteins. We followed that provided from the sequence homology analysis of the different Pennycress sequences with the TAIR and UNIPROT databases (**Supplementary Table S2**). This nomenclature was similar to that followed in Umate (2012) in their comparative analysis of oleosins, caleosins and stereolisins in different plant species. Our analysis revealed the presence of 21 genes encoding oleosin-related proteins in the Pennycress genome. Their number was higher than that obtained in Arabidopis (17 oleosin-related genes; Siloto et al., 2006; Chen et al., 2019) and smaller to that previously reported in other brassicaceae with high seed oil content like *Brassica napus*, in which 65 oleosin related genes were identified in the BLAST search (Chen et al., 2019).

**Table 1:** Genes encoding proteins involved in oil body formation in the Pennycress (*Ta*) genome.

	Protein name	*Ta* chr.	Gene position	*Ath* Gene	gene length	CDS length	*Ta* predicted protein	% identitty with *Ath*	aaprot *Ta*	aa*Ath*	RNAseq
Oleosin	OLE1 (SL)	OU466863.1	TAV2_LOCUS24479	AT4G25140	682	534	CAH2078007.1	88.37%	177	173	✓
	OLE2(SH)	OU466863.1	TAV2_LOCUS23202	AT5G40420	945	636	CAH2080051.1	81.82%	211	199	✓
	OLE3 (SL)	OU466862.2	TAV2 LOCUS18878	AT5G51210	572	441	CAH2070485.1	79.14%	146	141	✓
	OLE4(SH)	OU466858.1	TAV2_LOCUS5704	AT3G27660	963	597	CAH2045152.1	73.94%	198	191	✓
	OLE5(SH)	OU466859.2	TAV2_LOCUS10168	AT3G01570	692	579	CAH2055378.1	87.91%	192	183	✓
	OLE6(U)	OU466857.2	54339184 - 54339675	AT1G48990	492	492		76.07%	163	169	
	OLE8(U)	OU466859.2	TAV2_LOCUS9131	AT3G18570	495	495	CAH2053377.1	85.98%	164	166	✓
	OLET1	OU466862.2	67477510 -67478454	AT5G07560	945	426		65.96%	142	153	✓
	OLET2	OU466862.2	67479738 -67480612	AT5G07550	875	336		71.43%	112	106	✓
	OLET3	OU466862.2	67481618 -67482195	AT5G07550	578	330		72.64%	109	106	
	OLET4	OU466862.2	TAV2_LOCUS21826	AT5G07540	1130	657	CAH2073673.1	49%	218	190	
	OLET5	OU466862.2	TAV2_LOCUS20349	AT5G07530	1686	1272	CAH2073675.1	47.42%	423	543	✓
	OLET6	OU466862.2	TAV2_LOCUS20350	AT5G07530	798	678	CAH2073677.1	48.56%	225	512	✓
	OLET7	OU466862.2	67491755 -67493913	AT5G07530	944	561		32%	187	543	
	OLET8	OU466858.1	2474245 - 2472747	AT5G61610	1499	678		61.88%	225	294	✓
	OLET9	OU466859.2	TAV2_LOCUS8821	AT5G07530	916	444	CAH2052429.1	46.97%	147	543	
	OLEX1	OU466862.2	3169386-3169826	AT5G56100	441	441		71.23%	146	150	
	OLEX2	OU466863.1	TAV2_LOCUS24297	AT5G56100	752	630	CAH2077873.1	38.85%	209	150	✓
	Ø1OLE3	OU466862.2	13357891-13358103	AT5G51210				77.59%	70		
	Ø20LE3	OU466862.2	13425066-13424836	AT5G51210				76.19%	70		
	Ø3OLE3	OU466862.2	13444620 - 13444928	AT5G51210				57.78%	90		
OBAP	OBAP1a	OU466857.2	TAV2_LOCUS320	AT1G05510	918	726	CAH2038840.1	95.85%	241	241	✓
	OBAP1b	OU466860.2	7617497-7618636	AT2G31985	1140	696		92.61%	231	241	✓
	OBAP2a	OU466858.1	9714517-9715989	AT5G45690	1473	744		85.02%	247	247	✓
	OBAP2a2	OU466859.2	10201581 -10203055	AT5G45690	1475	733		69.59%	210	247	
	OBAP2b	OU466863.1	TAV2_LOCUS23394	AT4G18920	1120	744	CAH2078585.1	94.74%	247	247	✓
	OBAP2c	OU466857.2	4134539-4135532	AT1G29680	994	714		86.50%	237	237	✓
SEIPIN	SEIPIN1	OU466862.2	TAV2_LOCUS22247	AT5G16460	1203	1065	CAH2072608.1	79.26%	354	368	✓
	SEIPIN2	OU466857.2	TAV2 LOCUS2470	AT1G29760	1790	1632	CAH2034380.1	73.75%	543	526	✓
	SEIPIN3	OU466860.2	6317412-6319945	AT2G34380	2534	1650		74.49%	549	509	✓

Gene position and comparison with their Arabidopsis (*Ath*) orthologs is also shown. Their identification in the RNA-Seq analysis is also included.

**Figure 1:**
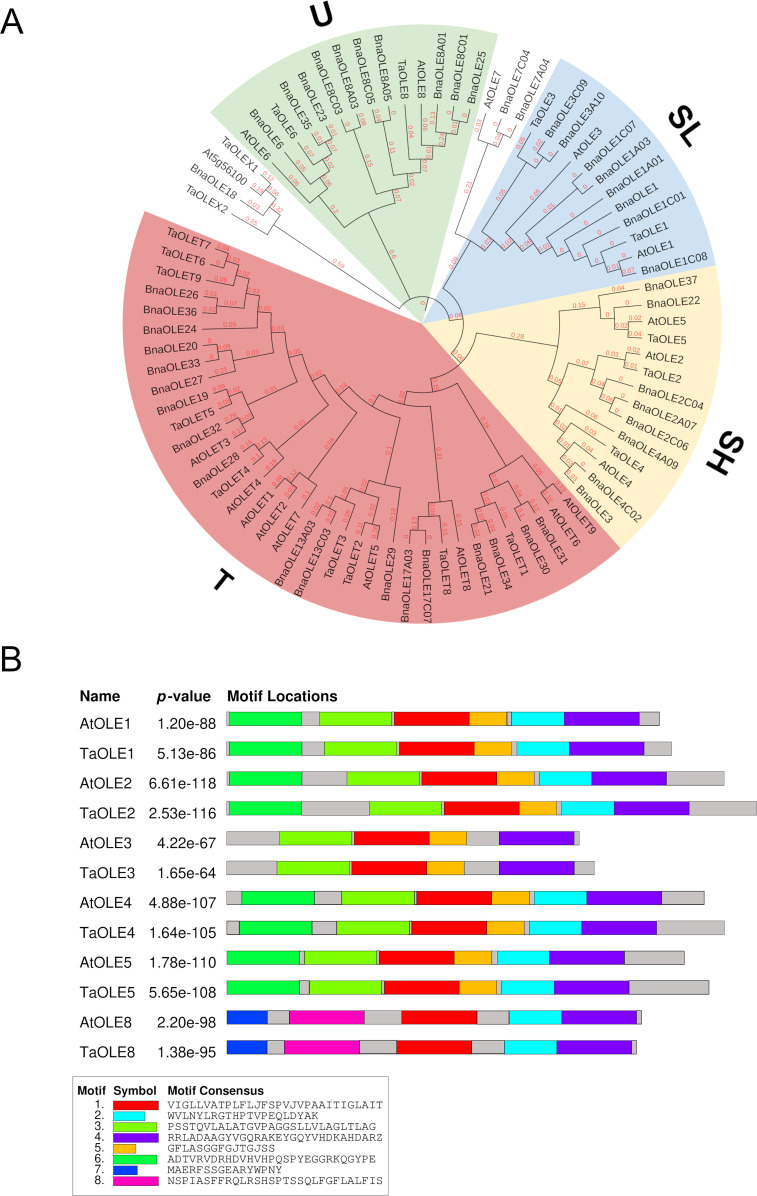
Phylogenetic tree of *OLE* genes from Pennycress and motif sequence analysis. **(A)** Phylogenetic analysis of *OLE* genes in plants. Orthologs of the Pennycress OLE (*TaOLE*) genes from *Arabidopsis thaliana* (At) and *Brassica napus* (Bna) were searched from databases and analyzed according to methods section. Separation of *OLE* genes into U (Universal), T (Tapetum), SL (Seed Low molecular weight) and SH (Seed High molecular weight) lineages is marked in different colors. **(B)** Common motifs shared between Pennycress and Arabidopsis OLE proteins. Motif consensus sequence is highlighted in the box.

Four out of the five oleosin (P, U, SL, SH and T) lineages were identified in the Pennycress genome in our analysis. **Figure 1** shows an un-rooted phylogenetic tree in which the 21 identified Pennycress oleosin genes were analyzed together with the Arabidopsis and *Brassica napus* ones. The phylogenetic analysis showed that T-type oleosins were the largest clade, while the rest of the lineages showed similar members (**Figure 1A**). Type S oleosins are seed-specific and, therefore, those directly involved in seed oil accumulation (Jolivet et al., 2009; Huang and Huang, 2015; Huang, 2018; Chen et al., 2019). Two different S-subtype oleosins can be found in plants, SH and SL (Huang and Huang, 2017). In our analysis, *Ta*OLE1 and *Ta*OLE3 grouped with other SL oleosins from Arabidopsis or *Brassica napus*, while *Ta*OLE2, *Ta*OLE4 and *Ta*OLE5 were grouped with SH (seed high molecular weight) ones (**Table 1**; **Figure 1A**). All these genes contained the oleosin family domain PF01277 (Pfam database) and IPR000136 (Interpro database) when checked in the Interpro (https://www-ebi-ac.uk/interpro/) analysis of classification of protein family. A common hallmark in all type S oleosin genes from Brassicaceae is the presence of a single intron (Huang, 2018) that was also detected in the *TaOLE* genes. Pennycress oleosin proteins had the same structure than those from other species; a hydrophyllic N-terminal region, a central hydrophobic hairpin of 72 residues and an amphipatic C-Terminal domain (Huang, 2018; Chen et al., 2019). The proline knot motif (PX_5_SPX_3_P), typical in the central part of the hairpin, was also detected in the Pennycress *Ta*OLE proteins (Huang, 2018; Chen et al., 2019). This motif is essential for oleosin targeting to the ER or OB (Huang and Huang, 2017). The motif-based sequence analysis (MEME) identified six conserved motifs in Pennycress *Ta*OLE1, *Ta*OLE2, *Ta*OLE4 and *Ta*OLE5, five conserved motifs in *Ta*OLE8 and four conserved ones in *Ta*OLE3, the shortest one, with respect to their orthologs in Arabidopsis (**Figure 1B**) or *Brassica napus* (**Supplementary Figure S1**), indicating that OLE proteins are highly conserved in Brassicaceae. In terms of protein sequence, Pennycress type S *Ta*OLE proteins shared a percentage identity with respect to Arabidopsis ranging from 73,9% (in the case of *Ta*OLE4) to 88,4% (in the case of *Ta*OLE1), **Table 1**. Interestingly, Pennycress *Ta*OLE4 protein showed a higher percentage of identity (80%) when compared to the *Bn*OLE4 protein. All oleosin proteins had molecular masses lower than 25 kDa, being *Ta*OLE2 the biggest one (**Table 1**). These results indicated that SH and SL oleosins from Pennycress were very similar to those previously analyzed in Arabidopsis or other Brassicaceae (Huang and Huang, 2015; Siloto et al., 2006; Chen et al., 2019).

Type U oleosins are present in all land plants (Huang, 2018; Chen et al., 2019). Our blast search only revealed two genes encoding type U oleosins in the Pennycress genome, *TaOLE6* and *TaOLE8* (**Table 1**; **Figure 1A**). This oleosin U-type has a characteristic AAPGA sequence in the C-terminal domain that was detected in both Pennycress *Ta*OLE6 and *Ta*OLE8 proteins as well as the characteristic lack of introns in their gene sequence. Interestingly, no protein with homology to the Arabidopsis *At*OLE7 was detected in the Pennycress genome in our analysis. Finally, T-type oleosins are a group characteristic of Brassicaceae and are mainly expressed in the flower tapetum (Huang, 2018). In the Arabidopsis genome, nine T-type oleosins are detected, all of them located in chromosome 5 in a tandem conformation except *TaOLET8* that is more distant in the same chromosome sequence (Huang and Huang, 2015). Our search revealed nine genes encoding T-type oleosins in the Pennycress genome (**Table 1**; **Figure 1A**). Similarly to Arabidopsis, most of these T-type oleosins were located in chromosome 6 in Pennycress, except *TaOLET9* and *TaOLET8* that were located in chromosome 3 and chromosome 2, respectively (**Supplementary Figure S2A**). In fact, Pennycress *Ta*OLET8 showed a high sequence identity with the Arabidopsis *Ta*OLET8 protein (62%). The arrangement of the T oleosin cluster in chromosome 6 is quite similar to that of Arabidopsis on chromosome 5 (**Supplementary Figure S2A**). It is important to mention that in the chromosome 6 of Pennycress, about 54Mb downstream of *TaOLET1*, we detected the *TaOLE3* gene (**Supplementary Figure S2A**). Upstream the *TaOLE3* gene sequence, we identified three tandem sequences very similar to the *TaOLE3* gene. We classified them as *OLE3* pseudogenes due to the presence of early stop codons that would give rise to much smaller proteins (⦰1OLE3, ⦰2OLE3, ⦰3OLE3; **Table 1**).

Finally, two genes that we named *TaOLEX1* and *TaOLEX2* were also identified in our blast search (**Table 1**). These two sequences showed homology to the Arabidopsis *At5g56100* (*OLEX1*) and *BnaOLE18* (**Figure 1**). It is worth mentioning that the genomic loci *AT5G56100* in *A. thaliana* was not included in the list of oleosins as it was annotated as glycine-rich protein oleosin domain (Umate, 2012).

### Expression of *TaOLE* genes during Pennycress seed maturation

We focused our analysis in genes encoding members of the seed specific type (SH and SL type, *TaOLE1*, *TaOLE2*, *TaOLE3*, *TaOLE4* and *Ta*OLE5), as well as *TaOLE8*, a U-type oleosin. On one hand, we used the RNA-Seq data (FPKM values) to monitor gene expression at the different stages of seed maturation (G, GY, YG, Y and M), covering the whole seed maturation process (Claver et al., 2024). On the other hand, we performed a qPCR analysis on samples from each maturation stage of the selected genes to contrast the RNA-Seq results.

**Figure 2:**
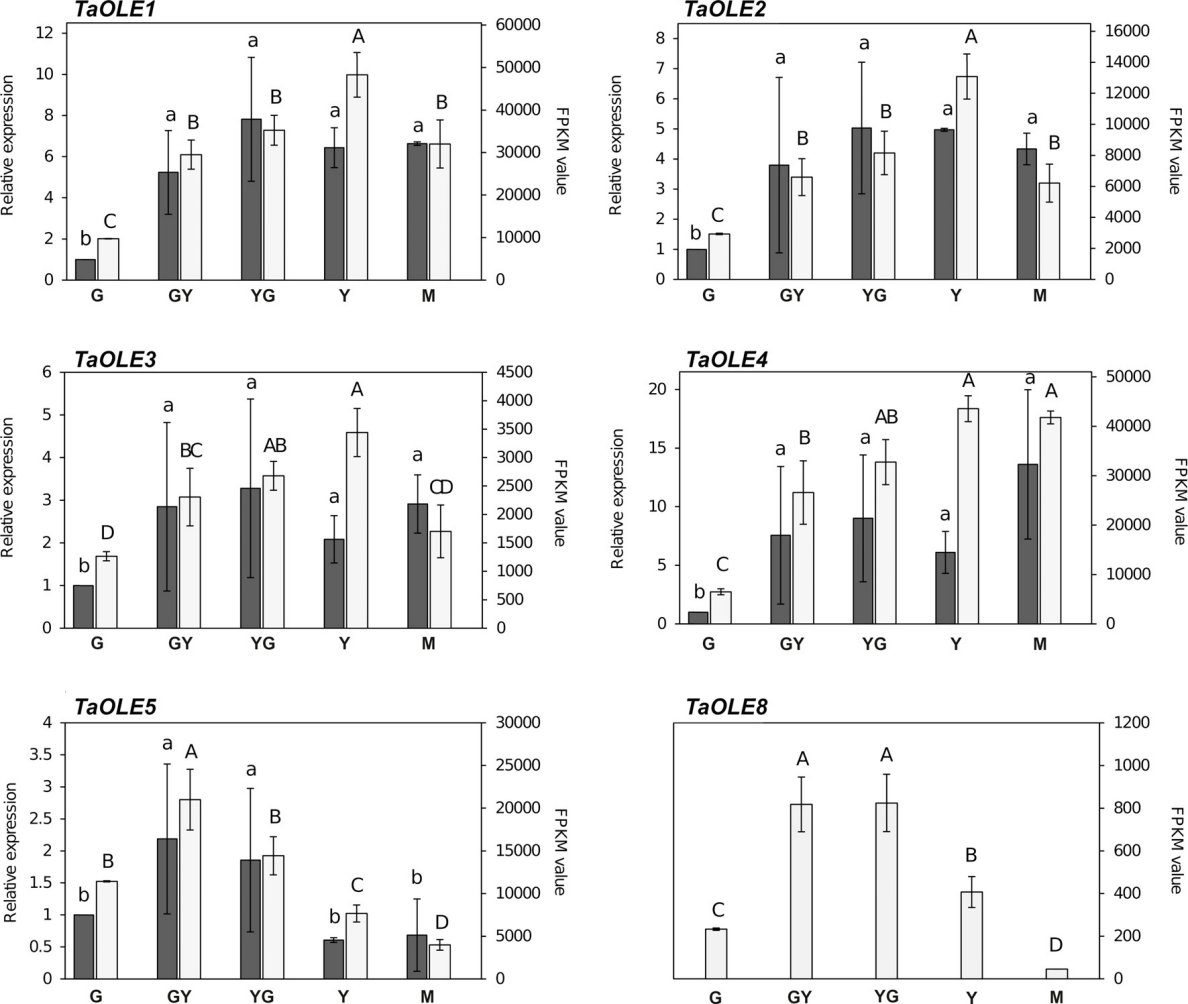
Expression of *TaOLE* genes during Pennycress seed maturation. Expression profiling of *TaOLE* genes by qPCR (grey bars) and RNA-Seq (white bars). For RNA-Seq data, expression levels are represented by FPKM values (right y-axis). Left y-axis represents qPCR relative expression data. The genes analyzed (*TaOLE1*, *TaOLE2, TaOLE3, TaOLE4, TaOLE5 and TaOLE8*) are indicated in each figure. Note the different scales on the *y* axes for different genes. For qPCR analysis, data were obtained from three independent pools of seeds from five plants of each line. For each maturation stage, Ct values were normalized against *ACT2* and *EF2α* housekeeping genes and referred to the G maturation stage for relative expression (fold-change). Data represent means ± SD of at least three biological replicates. Different lowercase (qPCR) and uppercase (RNA-Seq) letters show significant differences calculated by ANOVA among the different developmental stages during seed maturation of Pennycress (P < 0.05).

Expression of oleosin genes is shown in **Figure 2**. In general, the RNA-Seq data showed that genes encoding oleosins of the S-type increased their expression during Pennycress seed maturation, with their higher expression values at the YG-Y maturation stages, coinciding with the highest TAG accumulation (Claver et al., 2024) and oil droplet formation. An exception to this pattern was *TaOLE5* that showed its maximum gene expression at the GY stage, then decreasing in the later maturation stages (**Figure 2**). FPKM values indicated that *TaOLE1, TaOLE4* and *TaOLE5* were the most abundant oleosins at the initial G stage (**Figure 2**). Upon seed maturation, *TaOLE1*, *TaOLE2*, *TaOLE3* and *TaOLE4* genes increased their expression between 3 to 8-fold from the G to the Y stage (**Figure 2**). In fact, FPKM values indicated that mRNAs from *TaOLE1* and *TaOLE4* were the most expressed ones in all stages during Pennycress seed maturation (**Figure 2**). Expression of the U-type *TaOLE8* gene showed also a 3,5-fold increase of mRNA levels upon Pennycress seed maturation, showing maximum expression at the GY and YG stages, before that of the *TaOLE1*-*TaOLE4* genes. However, its FPKM values were the lowest of all the *OLE* genes analyzed (**Figure 2**). Finally, expression of T-type oleosins like *TaOLET1*, *TaOLET2*, *TaOLET5*, *TaOLET6* and *TaOLET8* was also detected in the RNA-Seq analysis. Again, their absolute FPKM values indicated a much lower expression (3-4 orders of magnitude) with respect to the SL and SH-type oleosins in the Pennycress seed, suggesting that they might not have a relevant role in seed-oil accumulation and oil body formation in Pennycress (**Supplementary Figure S2B**).

### Genome-wide identification of *TaSEIPIN* family genes in the Pennycress genome

**Figure 3:**
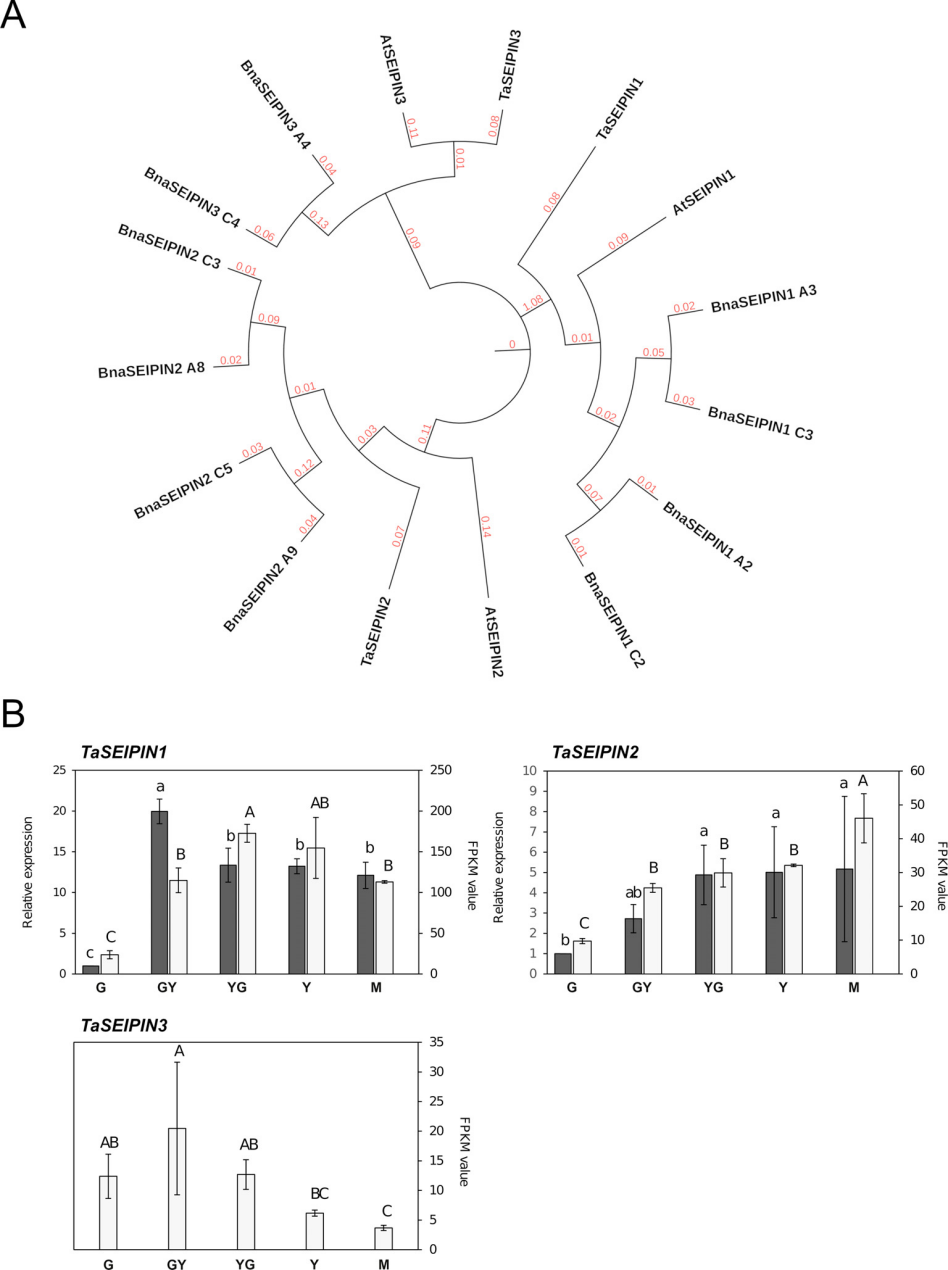
Phylogenetic analysis and gene expression of *TaSEIPIN* genes from Pennycress. **(A)** Phylogenetic analysis of *SEIPIN* genes from Pennycress (Ta). Orthologs of the Pennycress *SEIPIN* genes from *Arabidopsis thaliana* (At) and *Brassica napus* (Bna) were searched from databases and analyzed according to methods section. **(B)** Expression profiling of *TaSEIPIN* genes by qPCR (grey bars) and RNA-Seq (white bars). For RNA-Seq data, expression levels are represented by FPKM values (right y-axis). Left y-axis represents qPCR relative expression data. The genes analyzed (*TaSEIPIN1*, *TaSEIPIN 2*, and *TaSEIPIN3*) are indicated in each figure. For qPCR analysis, data were obtained from three independent pools of seeds from five plants of each line. Note the different scales on the *y* axes for different genes. For qPCR analysis, data were obtained from three independent pools of seeds from five plants of each line. For each maturation stage, Ct values were normalized against *ACT2* and *EF2α* housekeeping genes and referred to the G maturation stage for relative expression (fold-change). Data represent means ± SD of at least three biological replicates. Different lowercase (qPCR) and uppercase (RNA-Seq) letters show significant differences calculated by ANOVA among the different developmental stages during seed maturation of Pennycress (P < 0.05).

Seipin proteins play an important role in OB formation in humans and yeast (Salo et al., 2020). While a single *SEIPIN* gene exists in yeast or animals, three *SEIPIN* genes were identified in the Arabidopsis genome (Cai et al., 2015). Our search for *SEIPIN* genes in the Pennycress genome resulted in the identification of three putative *TaSEIPIN* genes with high homology with respect to their Arabidopsis orthologs (**Table 1**; **Figure 3A**). In terms of protein identity, Pennycress seipin proteins showed a 79.26, 73.75 and 74.49% identity with respect to Arabidopsis SEIPIN1, SEIPIN2 and SEIPIN3 proteins, respectively (**Table 1**; **Figure 3A**). Pennycress seipin proteins showed higher identity with seipin proteins from *Brassica napus*, another high seed oil accumulating species (**Figure 3A**). The MEME analysis showed three conserved motifs in Pennycress *Ta*SEIPIN1, and five conserved ones for *Ta*SEIPIN2 and *Ta*SEIPIN3, respectively with respect to their Arabidopsis orthologs (**Supplementary Figure S3**).

### Expression of *TaSEIPIN* genes during Pennycress seed maturation

The expression pattern of *TaSEIPIN* genes was monitored through Pennycress seed maturation. All the three *SEIPIN* genes were identified in the RNA-Seq analysis, and specific primers allowed to monitor their expression by qPCR. FPKM values for each of the three *TaSEIPIN* genes indicated that *TaSEIPIN1* was the most abundant one (ten-fold) in the Pennycress seeds (**Figure 3B**). Their expression patterns also differed. *TaSEIPIN1* mRNA levels increased rapidly from G to the GY and YG seed maturation stages, then keeping its expression levels in the rest stages of seed maturation in the RNA-Seq data (**Figure 3B**). In the qPCR analysis, *TaSEIPIN1* mRNA levels peaked at the GY stage, also maintaining high expression values in the rest of the stages (**Figure 3B**). This higher expression at the GY-YG maturation stages might be consistent with previous data of the role of SEIPIN1 in TAG accumulation in Arabidopsis (Cai et al., 2015). *TaSEIPIN2* gene mRNA levels increased gradually from G to YG/Y stages, maintaining their mRNA levels at the later Y and M stages (**Figure 3B**). Nevertheless, FPKM values indicated that in the YG or Y stages, expression of *TaSEIPIN1* was 5-6 fold higher than that of *TaSEIPIN2* (**Figure 3B**). *TaSEIPIN3* gene showed the lowest expression values of all the *TaSEIPIN* genes, with maximum expression between the G and YG stages (**Figure 3B**).

### Genome-wide identification of *TaOBAP* family genes in the Pennycress genome

**Figure 4:**
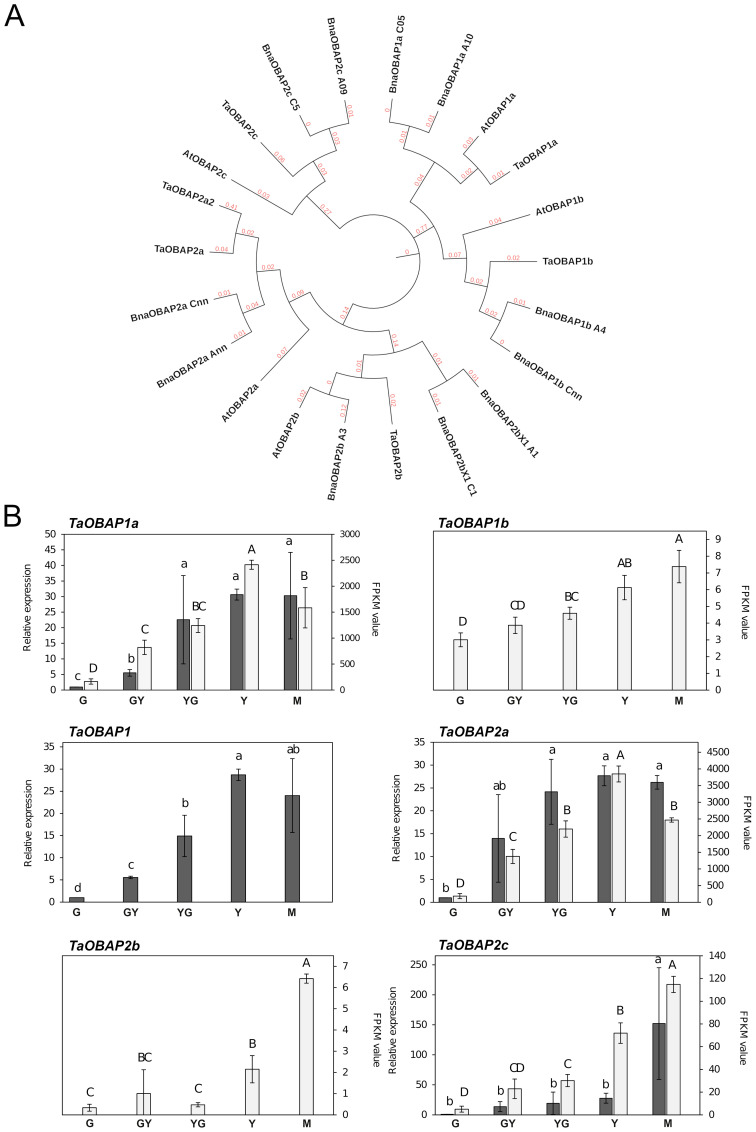
Phylogenetic analysis and gene expression of *TaOBAP* genes from Pennycress. **(A)** Phylogenetic analysis of *OBAP* genes from Pennycress (Ta). Orthologs of the Pennycress *TaOBAP* genes from *Arabidopsis thaliana* (At) and *Brassica napus* (Bna) were searched from databases and analyzed according to methods section. **(B)** Expression profiling of *TaOBAP* genes by qPCR (grey bars) and RNA-Seq (white bars). For RNA-Seq data, expression levels are represented by FPKM values (right y-axis). Left y-axis represents qPCR relative expression data. The genes analyzed (*TaOBAP1a*, *TaOBAP1b*, *TaOBAP1*, *TaOBAP2a*, *TaOBAP2b*, *TaOBAP2c*) are indicated in each figure. Note that *TaOBAP1b* and *TaOBAP2b* data were obtained only from RNA-Seq data. *TaOBAP1* show expression (q-PCR) of both *TaOBAP1a* and *TaOBAP1b* isoforms. For qPCR analysis, data were obtained from three independent pools of seeds from five plants of each line. Note the different scales on the *y* axes for different genes. For qPCR analysis, data were obtained from three independent pools of seeds from five plants of each line. For each maturation stage, Ct values were normalized against *ACT2* and *EF2α* housekeeping genes and referred to the G maturation stage for relative expression (fold-change). Data represent means ± SD of at least three biological replicates. Different lowercase (qPCR) letters and uppercase (RNA-Seq) letters show significant differences calculated by ANOVA among the different developmental stages during seed maturation of Pennycress (P < 0.05).

Genes encoding OBAP proteins can be identified in the genomes of many plant species, including monocots, dicots, conifers, primitive plants, mosses and even some algae (López-Ribera et al., 2014; Huang, 2018). Their role is still not well understood. They lack hydrophobic motifs allowing their insertion into OBs, suggesting that they should not play an important structural role (López-Ribera et al., 2014). With respect to their genome distribution, many dicots contain a variable number of genes distributed in two subfamilies (López-Ribera et al., 2014). The Arabidopsis genome contained five *AtOBAP* genes, distributed in two subfamilies, subfamily 1 (*AtOBAP1a* and *AtOBAP1b*) and three genes of subfamily 2 (*AtOBAP2a*, *AtOBAP2b* and *AtOBAP2c*; **Figure 4A**). Our search in the Pennycress genome revealed the existence of 6 *TaOBAP* genes, two of them *TaOBAP1a* and *TaOBAP1b*, with high homology (95,8 and 92,6%, respectively) with their Arabidopsis orthologs, (**Table 1**; **Figure 4A**). Other four *TaOBAP* genes could be identified in the Pennycress genome that grouped with subfamily 2 OBAPs (**Table 1**; **Figure 4A**). Genes encoding *TaOBAP2b* and *TaOBAP2c* showed high homology with their Arabidopsis orthologs (**Table 1**). Interestingly, two different loci were identified in the Pennycress genome, encoding a protein with homology to the OBAP2a protein from Arabidopsis or *Brassica napus* (**Table 1**; **Figure 4A**). We named *Ta*OBAP2a to the isoform with higher identity (85.02%) with the Arabidopsis *AtOBAP2a* gene (*At5g*45690) and *Ta*OBAP2a2 to that showing lower homology (69.59%) with respect to the Arabidopsis one (**Table 1**). The MEME analysis showed very similar conserved motifs of the different Pennycress OBAP proteins when compared with their Arabidopsis orthologs with except for the Pennycress *Ta*OBAP1b, in which the fourth domain present in the C-terminal region of the Arabidopsis *At*OBAP1b protein, was absent in the Pennycress one (**Supplementary Figure S4A**). Similar comparison with *Brassica napus* OBAPs also showed high motif conservation except *Ta*OBAP2b with *Bn*OBAP2b_A3 (but not with *Bn*OBAP2b_X1_C1 or _A1) and *Ta*OBAP2c with *Bn*OBAP2c_A09 (but not with *Bn*OBAP2c_C5), (**Supplementary Figure S4B**).

### Expression of *TaOBAP* genes during Pennycress seed maturation

The expression pattern of *TaOBAP* genes was monitored through Pennycress seed maturation. All the *TaOBAP* genes were detected in the RNA-Seq analysis, with the exception of *TaOBAP2a2*. qPCR was performed whenever possible, limited by the high homology among *TaOBAP* genes that difficulted the design of optimal primers to monitor the expression of each specific gene separately. In those cases, primers monitoring *Ta*OBAP gene expression from subfamily 1 or 2 separately were performed. The results are shown in **Figure 4B**. In all cases, mRNA levels detected in the RNA-seq analysis showed an increase during Pennycress seed maturation from G with maximum expression at the Y and M stages (**Figure 4B**), consistent with the highest TAG accumulation. These results were confirmed by qPCR (**Figure 4B**). The FPKM values for each of the *TaOBAP* genes analyzed indicated that *TaOBAP1a* and *TaOBAP2a* were the highest expressed *TaOBAP* genes during Pennycress seed maturation, showing the rest of the genes much lower expression values (**Figure 4B**). This pattern was consistent to that previously reported for the maize *OBAP1* gene (López-Ribera et al., 2014). It is worth mentioning that the absolute FPKM values of *TaOBAP* genes indicated that expression of *TaOBAP1a* or *TaOBAP2a* genes was at least one order of magnitude less with respect to the most expressed oleosin genes (*TaOLE1* and *TaOLE4*; **Figure 2**). However, their relative increase seemed to be much higher than oleosins, at least at the transcript level (15-30 fold at the Y stage for *TaOBAP1a*).

### Expression of other genes encoding oil body related proteins during Pennycress seed maturation

**Figure 5:**
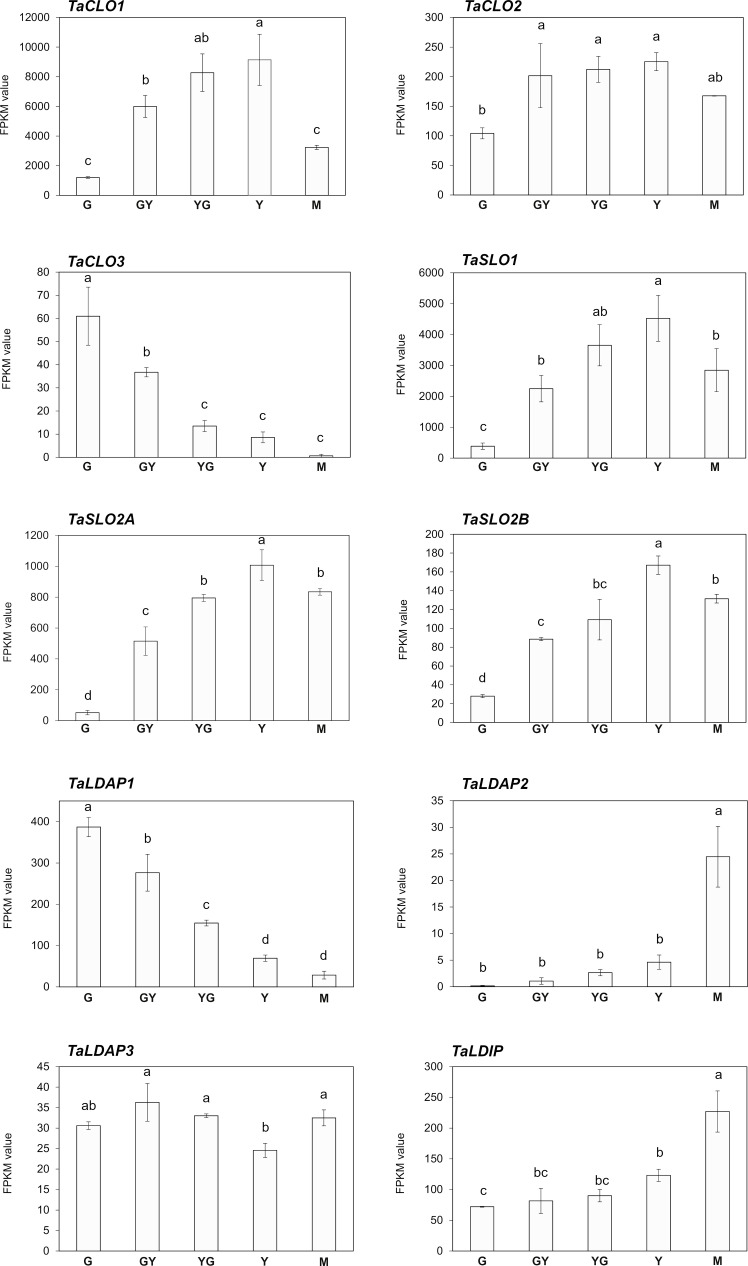
Expression of caleosin (*TaCLO*), stereolisin (*TaSLO*), lipid droplet associated proteins (*TaLDAP*) and lipid droplet interacting protein (*TaLDIP*) during Pennycress seed maturation. RNA-Seq data, expression levels are represented by FPKM values (left y-axis). The different genes analyzed (*TaCLO1*, *TaCLO2*, TaCLO3, *TaSLO1*. *TaSLO2A*, *TaSLO2B*, *TaLDAP1*, *TaLDAP2*, *TaLDAP3*, and *TaLDIP*) are indicated in each figure. Note the different scales on the *y* axes for different genes. Data represent means ± SD of at least three biological replicates. Different uppercase (RNA-Seq) letters show significant differences calculated by ANOVA among the different developmental stages during seed maturation of Pennycress (P < 0.05).

Oleosins, SEIPINs and OBAPs are not the only proteins present in plant OBs. Caleosins (CLOs) and stereolisins (SLOs) are also detected in high amounts (Baud et al., 2009; Jolivet et al., 2011; Liu et al., 2022; Huang, 2018; Miklaszewska et al., 2023). Caleosins (CLO) have been related with stress responses (Liu et al., 2022), while stereolisins (SLO) seem to be involved in OB mobilization during germination, making accessible the TAG to lipases and favoring germination (Jolivet et al., 2004; Baud et al., 2009). Our search in the Pennycress genome revealed three *TaCLO* genes (*TaCLO1*, *TaCLO2* and *TaCLO3*) encoding proteins with high homology to their Arabidopsis orthologs (90.20, 88.48 and 88.56, respectively; **Supplementary Table S3**). Analysis of *TaSLO* genes revealed the existence of three genes, one encoding a *TaSLO1* type with high homology to the Arabidopsis *SLO1* gene (**Supplementary Table S3**) and two different loci encoding two different *Ta*SLO2 stereolisins, named *TaSLO2A* and *TaSLO2B*, with different homology with respect to the single Arabidopsis *SLO2* gene (90.79 and 77.26, respectively; **Supplementary Table S3**). Analysis of the RNA-Seq data for *TaCLO* and *TaSLO* genes showed a similar pattern of expression during Pennycress seed maturation (**Figure 5**) to that of the *TaOLE* genes (**Figure 2**). *TaSLO* and *TaCLO* genes increased their mRNA levels from G to Y/M stages, with the only exception of *TaCLO3*, whose expression was higher at the G stage and then decreased to almost undetectable values at the M one (**Figure 5**).

Finally, three *TaLDAP* genes (*TaLDAP1*, *TaLDAP2* and *TaLDAP3*) were detected in the Pennycress genome with high homology with respect to their Arabidopsis orthologs (76,79%, 79,13% and 94,69%, respectively). All three genes were detected in the RNA-Seq analysis, although their expression values were two-three orders of magnitude lower than those obtained for *TaOLE* or *TaOBAP* genes or even some *TaCLO* or *TaSLO* genes (**Figure 5**). Their expression profiles also showed some differences. *TaLDAP1* decreased its mRNA levels to almost undetectable ones from the G to the Y or M maturation stages (**Figure 5**). On the contrary, *TaLDAP2* expression showed a complete opposite pattern increasing with seed maturation while *TaLDAP3* expression levels were not significantly modified during seed maturation (**Figure 5**). LDIP has been reported to cooperate with SEIPIN and LDAPs in the formation of LDs (Pyc et al., 2021). A single *TaLDIP* gene was detected in the Pennycress genome, highly homologous (79,52%) to its Arabidopsis ortholog. Its expression profile showed an increase in mRNA levels during seed maturation, with the highest expression values at the Y and particularly M late stages (**Figure 5**). Our expression data for *TaLDAP2*, *TaLDAP3* and *TaLDIP* are consistent with a recent work in which the role of LDAPs and LDIP protein was analyzed in Pennycress (Guzha et al., 2024).

### Characterization of Pennycress seed oil bodies at the protein level

**Figure 6:**
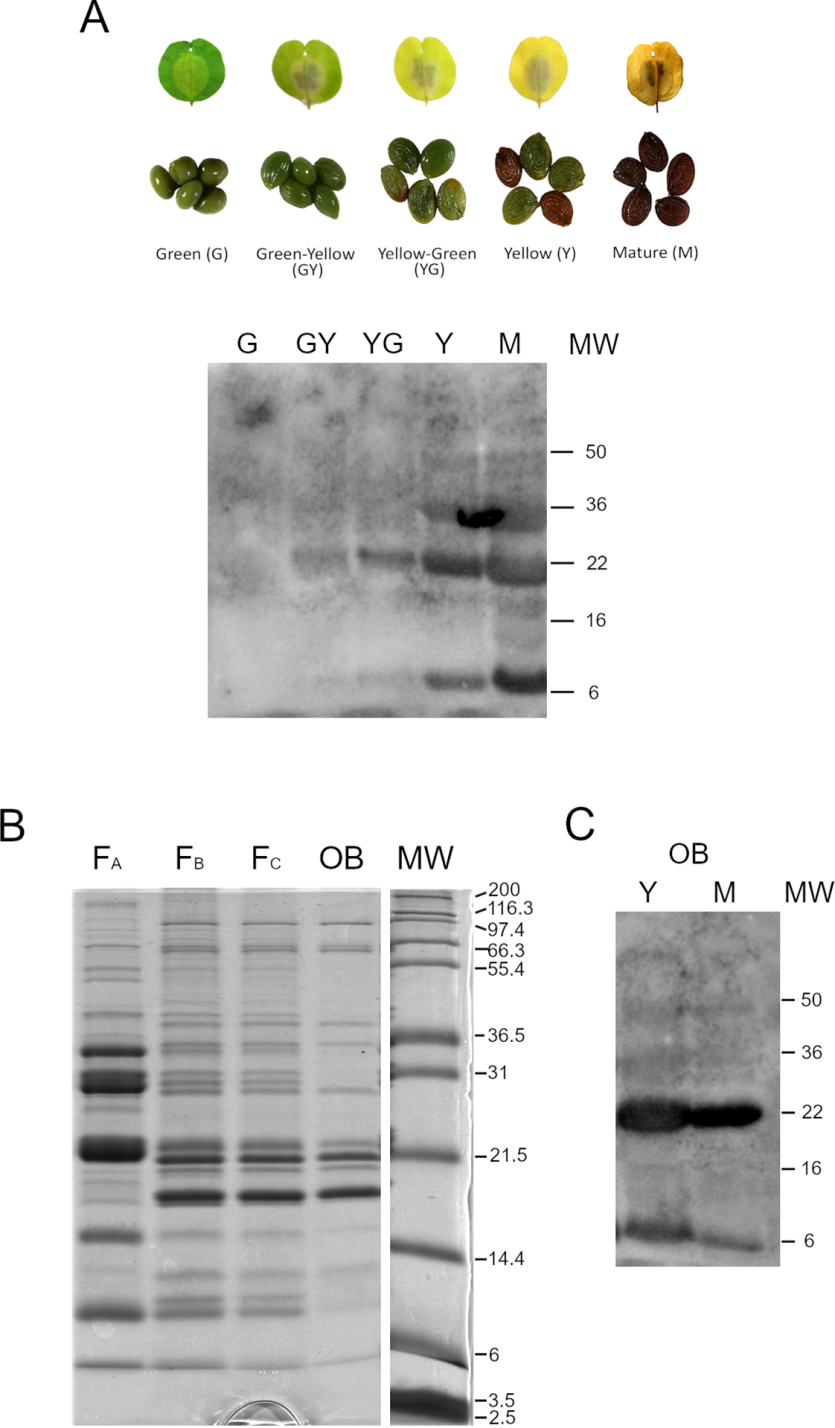
Characterization of Pennycress OBs at the protein level. **(A)** Western blot analysis of *Ta*OLE2 protein during Pennycress seed maturation. Protein extracts were obtained from the five seed maturation stages showed in the photograph above the western blot images. Ten micrograms of total protein were loaded per lane. **(B)** SDS-PAGE analysis of OB fractionation from total seed extracts from Y and M stages. **(C)** Western blot against the anti-OLE2 antibody of OB-enriched fractions. Four micrograms of total protein were loaded per lane.

We characterized Pennycress OBs at the protein level. Total protein fractions from seeds corresponding to the different maturation stages used for the transcriptomic analysis were obtained, separated by SDS-PAGE and blotted against antibodies of the OLE2 protein commercially available. Our results show that the OLE2 antibody cross-reacted with total seed Pennycress proteins. A band with an apparent size of 22 kDa reacted with the antiOLE2 antibody in the total protein extracts (**Figure 6A**). This size corresponded well with the expected size of the Pennycress *Ta*OLE2 protein (**Table 1**). A band of lower size (4-6 kDa) also cross-reacted with the OLE2 antibody (**Figure 6A**), probably corresponding to some protein degradation. The *Ta*OLE2 protein accumulation pattern corresponded well to its expression levels (**Figure 2**).

As a further step to a more detailed analysis of proteins present in the OBs, we obtained OB-enriched fractions from crude extracts from Pennycress seeds by subsequent fractionation steps as described in Methods section. These fractions were obtained from Y and M seed maturation stages, since any attempts to obtain OB-enriched fractions from earlier stages were unsuccessful. In each fractionation step, the floating fraction corresponding to OBs was recovered and subsequently fractionated in the presence of urea to eliminate as much as possible soluble proteins contaminating the OB fractions. This procedure is illustrated in **Figure 6B**. OB enriched fractions showed a protein composition that showed most of the proteins migrating in the 14-22 kDa region (F_B_; F_C_ and OB fractions; **Figure 6B**). Interestingly, bands migrating at higher or lower molecular weight zones were mostly eliminated with the urea treatment in the first fractionation step (**Figure 6B**, F_A_ to F_B_). It is worth mentioning that the final OB enriched fraction obtained showed a specific accumulation of proteins ranging from 22-17 kDa where oleosins were expected to migrate (**Figure 6B**). In fact, western blot analysis of OB-enriched fractions from both YELLOW and MATURE seed maturation stages showed high accumulation of the *Ta*OLE2 protein in both fractions from both stages (**Figure 6C**). Other protein bands were also detected. Thus, a protein band below the 31 kDa molecular marker was also observed, corresponding to the region where caleosins are expected to migrate (**Figure 6B**). Similarly, proteins above the 36,5 kDa marker are compatible with the expected size of stereolisins (**Figure 6B**).

**Figure 7:**
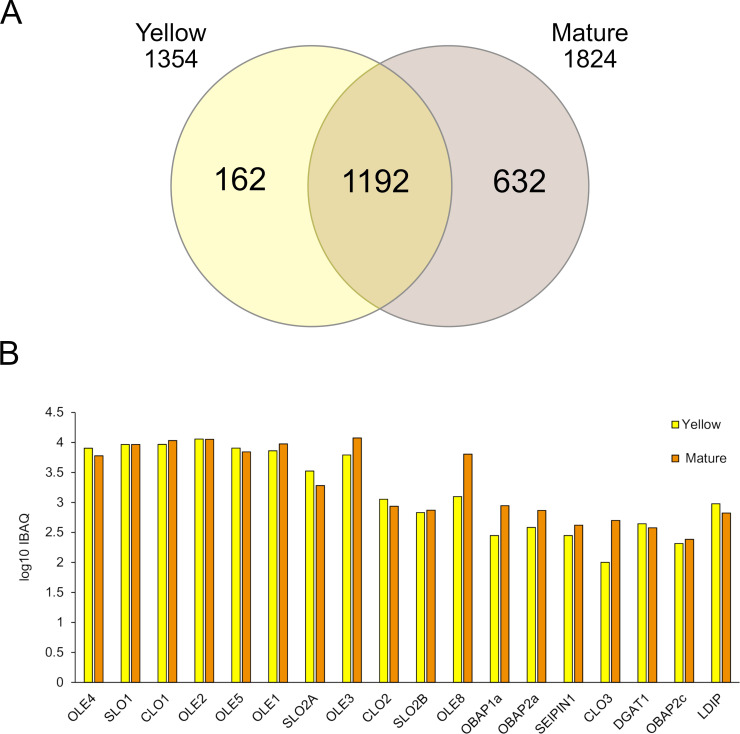
Proteomic analysis of OB-enriched fractions from YELLOW and MATURE seeds from Pennycress. **(A)** De Venn diagram showing the number of isolated proteins identified in the proteomic analysis in both maturation stages. **(B)** Log10iBAQ of OB-related proteins. Data were obtained from two independent biological replicates.

OB enriched fractions obtained from the Y and M maturation stages were subjected to proteomic analysis through LC-TIMS-MS/MS. Protein intensities were determined by the intensity-based absolute quantification (iBAQ). In total, we detected 1,986 proteins that matched with at least two peptides in the two biological replicates analyzed. Among them, 1,192 proteins were common in OB fractions from both seed maturation stages. 162 proteins were specific of the Y stage while 632 were specific of the M one (**Figure 7A**), indicating that the protein groups were higher in OB from the final seed maturation stage. The Pennycress accession number for each protein, their relative abundance parameters (raw data, average area) are shown in **Supplementary Table S4**.

All the OB structural proteins expected to be present in OB fractions were found in the common proteins of both stages. Among these proteins, oleosins were the most abundant ones as deduced from their iBAQ values (**Figure 7B**). All *Ta*OLE proteins identified in the proteome (*Ta*OLE1, *Ta*OLE2, *Ta*OLE3, *Ta*OLE4 and *Ta*OLE5) showed similar relative abundance between the Y and M OB fractions, with some small variations between both stages depending on the protein that might not have any biological significance (**Figure 7B**). This result was consistent with the results of the western-blot analysis of the *Ta*OLE2 protein in OB-enriched fractions (**Figure 5A**). *Ta*OLE8 protein was also detected, although its relative abundance was lower when compared to that from the *Ta*OLE1-*Ta*OLE5 proteins, particularly in the Y stage (**Figure 7B**). This lower relative abundance of *Ta*OLE8 could be consistent with its low mRNA levels, as shown in **Figure 2B**. It is also worth mentioning that the similar relative abundance of *Ta*OLE1, *Ta*OLE2, *Ta*OLE3, *Ta*OLE4 and *Ta*OLE5 proteins as detected in the proteomic analysis contrasted with the different FPKM values of the different *TaOLE* genes, as is the case of *TaOLE3*, with FPKM values much lower than the rest (**Figure 2B**) but with similar relative protein abundance (**Figure 7B**). This observation is similar to that reported by Miquel et al. (2014) when comparing gene expression and protein abundance of oleosins in Arabidopsis.

Other highly abundant proteins detected in the proteome analysis of OB enriched fractions from Pennycress seeds were stereolisins (*Ta*SLO1 and *Ta*SLO2A) and caleosin1 (*Ta*CLO1), (**Figure 7B**), that showed relative abundance values similar to those from oleosins. Caleosins are abundant proteins in the seed involved in the catabolism of reserve lipids upon seed germination, more concretely in lipophagy during LD breakdown (Liu et al., 2022; Miklaszewska et al., 2023). Similarly, stereolisins are highly abundant proteins in mature seeds, related with seed dormancy (Lin et al., 2002; Jolivet et al., 2004; Baud et al., 2009). It is worth mentioning that differently to Arabidopsis, two different *TaSLO2* genes were detected in the Pennycress genome and accordingly, two different *Ta*SLO2 proteins were found in the proteomic analysis. Interestingly, *Ta*SLO1 protein levels were higher than those from *Ta*SLO2A and *Ta*SLO2B proteins (**Figure 7B**) which might be consistent with the higher FPKM values of the genes (**Figure 5**). In general, the FPKM expression values correlated with the well iBAQ values of **Figure 7B**.

Other proteins detected as structural part of OBs are seipins and OBAPs. *Ta*SEIPIN1 was detected in the proteome analysis of OB enriched fractions from Pennycress seeds (**Figure 7B**), although its relative abundance was much lower than that of oleosins, caleosins or stereolisins in both seed maturation stages. The same occurred with *Ta*OBAP1a, *Ta*OBAP2a and *Ta*OBAP2c, which were also detected in the proteome from both OB enriched fractions at lower levels with respect to those from OLE proteins (**Figure 7B**). No *Ta*LDAP proteins were detected in the proteome of OB-enriched fractions from both Y and M stages, consistent with previous observations (Kretzschmar et al., 2020). However, a *Ta*LDIP protein was present in both stages, although it was a low abundant protein when compared with oleosins, *Ta*CLOs or *Ta*SLOs (**Figure 7B**). It is also worth mentioning that *Ta*DGAT1, the enzyme responsible for TAG biosynthesis through the Kennedy pathway and highly abundant in mature Pennycress seeds (Claver et al., 2024) was also present in the proteome from both OB enriched fractions (**Figure 7B**). It was not the only protein involved in fatty acid and lipid metabolism detected in the analysis. Other proteins involved in lipid and fatty acid biosynthesis like long-acyl chain synthetases (*Ta*LACS6, *Ta*LACS8 and *Ta*LACS9), the stearoyl ACP desaturase or proteins involved in VLCFA synthesis like the VLCFA reductase or the β-KCS18 synthase (*Ta*FAE1), were also detected in the analysis (**Supplementary Table S3**). This does not mean that these proteins were structural part of the OBs, but nevertheless they were detected in the enriched OB fractions analyzed.

## Discussion

Brassicaceae are a plant family characterized by a great heterogeneity in their seed oil content, with values ranging from 17 to 54%, depending on the species (Velasco et al., 1999; Oblath et al., 2016). Some of the members of the group have attracted the interest of biotechnologists because of the presence of unusual fatty acids, in addition to their high seed oil content, that are suitable for multiple non-edible applications. It is the case of *Ricinus communis* (accumulating ricinoleic acid; Bafor et al., 1991); *Camelina sativa* (accumulating high amounts of 18:3; Berti et al., 2016) or more recently Pennycress, that accumulates high amounts of erucic acid (Claver et al., 2017; Altendorf et al., 2019) and has become an emerging feedstock for biodiesel and biojet production (Moser, 2012; Claver et al., 2017). The reasons of these differences are poorly understood. Understanding the molecular and biochemical determinants of such differences in seed oil content and fatty acid composition among Brassicaceae might be of great interest for their application in plant breeding programs directed towards an improvement of seed oil content and characteristics. In that sense, our group identified differences in the Pennycress *Ta*FAE1 affinity for 20:1 CoA moieties with respect to that from Arabidopsis as a first explanation for the higher 22:1 presence in the Pennycress seed oil (Claver et al., 2020). More recently, the transcriptomic and lipidomic analysis during seed maturation showed a concerted contribution of the different TAG biosynthetic pathways explaining the high-erucic TAG accumulation in the Pennycress seed (Claver et al., 2024). However, not only TAG or VLCFA biosynthesis itself, but also TAG accumulation in OBs might be an additional control point of the high seed oil content and the different fatty acid composition of TAG in Pennycress. Although the role of LDAP and LDIP proteins in LD morphology has been studied in Pennycress (Guzha et al., 2024), the major proteins usually associated to OBs, like oleosins, caleosins or stereolisins, as well as their role in OB formation and stabilization, has not been studied in Pennycress. In this work we have analyzed at the genetic and protein levels, the different proteins associated to OBs in Pennycress, in an attempt to establish their specific roles in the oil storage machinery and their influence in the high seed oil content in this species.

Several proteins can be found associated with plant OBs, mainly oleosins, seipins, OBAPs, caleosins and stereolisins (Huang, 2018). These proteins have different roles in formation, stabilization and mobilization of OBs upon germination in seeds (Huang, 2018). Our genomic analysis revealed that genes encoding most of these proteins were detected in the Pennycress genome and that their gene structures and sequences were highly conserved with respect to Arabidopsis or *Brassica napus*, two species phylogenetically close to Pennycress with different seed oil content and fatty acid composition. 21 genes encoding oleosin related proteins (including three pseudogenes) were detected in the Pennycress genome (**Table 1**), a number closer to that from Arabidopsis (17 oleosin genes; Siloto et al., 2006) but lower to that from *Brassica napus* (65 oleosin genes; Chen et al., 2019). Therefore, the strategy of Pennycress to reach high seed-oil contents similar to those from *Brassica napus* is not based in the increase of oleosins participating in OB accumulation and stabilization, as seemed to be in the latter species. The same is true in the case of seipin or OBAP proteins. Seipins are key proteins for OB formation in eukaryotes (Cai et al., 2015; Taurino et al., 2018). Three *TaSEIPIN* genes were detected in the Pennycress genome with high homology to their three Arabidopsis orthologs (**Table 1**, **Figure 3A**, **Supplementary Figure S3**), showing conserved motif structures when compared with Arabidopsis or *Brassica napus* ones (**Supplementary Figure S3**). The role of OBAP proteins in OB formation and stabilization is poorly understood, being OBAP1 the only protein characterized to some extent in Arabidopsis (López-Ribera et al., 2014). Six *TaOBAP* genes were detected in the Pennycress genome. Interestingly, the *TaOBAP2a* gene showed two isoforms, *TaOBAP2a* and *TaOBAP2a2*, located in two different loci in the Pennycress genome, that were not detected in the genomes of Arabidopsis or Brassica (**Table 1**, **Figure 4A**, **Supplementary Figure S4**). However, we were not able to detect their expression individually in the RNA-Seq data or their presence in the proteomic analysis of OB enriched fractions, where a single *Ta*OBAP2a protein was detected (**Figures 5**, **7**; **Supplementary Table S2**). On the contrary, our analysis of the Pennycress genome detected two *TaSLO2* genes (*TaSLO2A* and *TaSLO2B)*, with individual expression profiles (**Figure 5**; **Supplementary Table S3**). Both *Ta*SLO2A and *Ta*SLO2B proteins were also individually detected in the proteomic analysis of enriched OB fractions from YELLOW and MATURE stages (**Figure 7**; **Supplementary Table S2**). These two different *TaSLO2A* and *TaSLO2B* genes represented a difference at the genomic level in Pennycress with respect to Arabidopsis. Our data suggest that, with some exceptions like *TaSLO2* or the apparent absence of a *TaOLE7* gene, the genes encoding proteins related with OB formation and stabilization are essentially similar in Pennycress with respect to other Brassicaceae with different seed oil content and fatty acid composition like Arabidopsis.

The analysis of gene expression and protein abundance of genes and proteins associated to OBs revealed some expression profiles and distribution patterns that provided some clues about their specific roles in OB formation and stabilization in Pennycress. In animal cells, the single seipin is critical for OB initiation, trapping neutral lipids in the ER bilayer and helping to the expansion of the OB (Salo et al., 2020). In Arabidopsis, SEIPIN1 seems to be involved with the initial steps of budding at the ER membrane and later OB formation (Cai et al., 2015; Taurino et al., 2018) although their mutational analysis suggested that they could also influence OB number and size (Cai et al., 2015). In Pennycress, the expression profile of both *TaSEIPIN1* and *TaSEIPIN3* genes, higher at the GY stage of seed maturation, may support a similar role in OB formation. FPKM values indicated that *TaSEIPIN1* mRNA levels were ten-fold more abundant than those from *TaSEIPIN3* (**Figure 3B**). Furthermore, only *Ta*SEIPIN1 was detected in the proteomic analysis of OB-enriched fractions from both Y and M maturation stages indicating that, similarly to Arabidopsis, *Ta*SEIPIN1 was the major seipin acting in Pennycress OBs and, because of its gene expression pattern, might have a role in OB formation. It is worth mentioning that the expression profile of *TaOLE* genes followed that of TAG accumulation in the seed during maturation (**Figure 2**) except for *TaOLE5*, that showed higher mRNA levels at the GY initial maturation stage, closer to those of *TaSEIPIN1* (**Figure 2**). FPKM values of the different *TaOLE* genes indicated that *TaOLE1*, *TaOLE4*, and *TaOLE5* were the most highly expressed genes during maturation of the Pennycress seed, with lower expression values of *TaOLE2* and particularly *TaOLE3* and *TaOLE8*, which were much lower than the rest (**Figure 2**). This expression profile, particularly that of *TaOLE5*, differed to some extent to that reported in Arabidopsis where *AtOLE1, AtOLE2* and *AtOLE4* were the most highly expressed during seed maturation while *AtOLE3* and *AtOLE5* were expressed at very low levels and no specific *AtOLE* gene showed an early expression pattern (Miquel et al., 2014; Mizzotti et al., 2018). The fact that *TaOLE5* showed the highest expression at the early stages of seed maturation, coinciding with those of *TaSEIPIN1* (**Figures 2**, **3**), might support a possible specific role of these two proteins at the initial stages of OB formation in Pennycress.

Pennycress OBs were also analyzed at the protein level through proteomics of OB-enriched fractions of the Y and M stages, since any attempts to obtain OBs from earlier fractions were not successful. Similar problems were found in Arabidopsis (Jolivet et al., 2011). Our proteomic data in Pennycress showed that *Ta*OLE1, *Ta*OLE2, *Ta*OLE3, *Ta*OLE4, and *Ta*OLE5 proteins were highly abundant in OB-enriched fractions, with no relevant differences in their relative abundance in both stages (**Figure 7**). Proteomic analysis of purified OB fractions has been performed in *Brassica napus* (a high seed oil containing species) and in Arabidopsis (with lower seed oil content when compared with Pennycress or Brassica) (Jolivet et al., 2004; 2009; 2011). *Brassica napus* is complex for this kind of proteomic analysis given that nine *Bn*OLE1, four *Bn*OLE2, two *Bn*OLE5, two *Bn*OLE4 and two *Bn*OLE3 proteins were detected (Jolivet et al., 2009). Nevertheless, the major band corresponding to oleosins showed the presence of two *Bn*OLE5, four *Bn*OLE4 and five *Bn*OLE1 proteins with no presence of *Bn*OLE3 (Jolivet et al., 2009; 2011). In their work in *Arabidopsis thaliana* WS ecotype, *At*OLE1 was the most abundant oleosin protein detected in oil bodies from mature seeds (Jolivet et al., 2004). *At*OLE4 and *At*OLE2 were detected in lower amounts when compared with *At*OLE1, while *At*OLE3 was hardly detected (Jolivet et al., 2004). However, protein analysis performed in seeds (not in purified OBs) in a Col0 Arabidopsis ecotype showed that all oleosins accumulated during embryo development and were present in mature seeds although *At*OLE5 (*Ta*OLE3) showed a 25% reduction in its abundance with respect to its maximal levels (Miquel et al., 2014; Deruyffelaere et al., 2015). In Pennycress all oleosins showed high abundance at the final maturation stages coinciding with the highest TAG accumulation (Claver et al., 2024). Our data, based in an integrative transcriptomic and proteomic analysis in OBs, point to a mechanism in which all the oleosin proteins might contribute to OB stabilization and TAG accumulation in Pennycress, helping to maintain such high seed-oil values. Jolivet et al. (2011) provided the first evidences that in *Brassica napus* there is a sequential deposition of integral OB proteins with differences between oleosins (*Bn*OLE5 or *Bn*OLE3 accumulating at 25 days after pollination or DAP (similar to our GY stage), while *Bn*OLE2 accumulated at 45 DAP, similar to our Y to M stages), caleosins (*Bn*CLO1 accumulating at 25 DAP) and stereolisins (very abundant at 45 DAP). Such a recruitment and the existence of interactions between OB proteins might facilitate the stability of OBs (Huang, 2018). Our expression data might be consistent with this hypothesis. Furthermore, our transcriptomic data suggest a role at the early stages of OB formation of both *Ta*SEIPIN1 and *Ta*OLE5 that could contribute to the formation and initial stabilization of OBs in Pennycress, respectively, allowing for the further recruitment of other oleosins upon seed maturation and OB formation. In that sense, it has been reported that in Arabidopsis, the LDIP protein interacts with SEIPIN1 to modulate LD formation (Pyc et al., 2021). In their recent work, Guzha et al. (2024) obtained *ldip* loss-of-function mutants in Pennycress that showed increased seed oil content. We detected a single *TaLDIP* gene in the Pennycress genome, showing the highest expression at the M stage (**Figure 5**). This high expression in the M stage is consistent with our proteomic data (**Figure 7**), suggesting that *Ta*LDIP in the Pennycress seed might also have a role in the late phases of OB accumulation.

The similar abundance of oleosin proteins in Pennycress OBs could be important for the control of OB size and number, and the recruitment of TAG with specific fatty acid compositions. Thus, Siloto et al. (2006) demonstrated in Arabidopsis that *AtOLE1* mutations resulted in changes in OB morphology, with *ole1* mutants showing larger OBs than the WT. These results were further confirmed in *Brassica napus* (Chen et al., 2019), showing that overexpression of *BnOLE* genes in Arabidopsis resulted in higher OB size except for those lines overexpressing *BnOLE1* (Chen et al., 2019). Given the results obtained in the *ole1* mutant (Siloto et al., 2006) and overexpressing *BnOLE1* lines (Chen et al., 2019), and without precluding a role of other OB-related proteins like SEIPIN or OBAPs, it is tempting to speculate with the hypothesis that the ratio between OLE1 (acting as a negative regulator of OB number and size), and the rest of the OLE proteins (acting as positive OB size controllers) could regulate OB size and their number in the Pennycress seed and therefore, be a key parameter for the control of the high oil content in the Pennycress seed. Moreover, while the *ole2* mutation did not modify the fatty acid composition in TAG, *ole1* mutations showed an increase in VLCFAs (20:1) in Arabidopsis (Siloto et al., 2006). Again, these results were consistent with previous data showing that the expression of *OLE* genes from Castor bean increased ricinoleic acid levels in TAG from transgenic Arabidopsis lines (Lu et al., 2009) or data from overexpressing *BnOLE* in Arabidopsis (Chen et al., 2019). Therefore, not only OB size and number, but also VLCFA composition of the TAG accumulated in OBs could also be related to the differences in OLE protein ratios in the sense that high OLE1/rest of OLE proteins ratios result in small OBs with low VCFA content while smaller OLE1/rest of OLE proteins ratios might result in larger OBs with high VLCFAs, which is the scenario occurring in Pennycress seed, as can be deducted from our proteomic data (**Figure 7**).

It is worth mentioning that oleosins are not the only proteins involved in the control of VLCFA-containing TAG recruitment to OBs. *clo1* and *clo2* mutants from Arabidopsis showed a decrease in 20:1 levels in OBs when compared to WT (Miklaszewska et al., 2023). Conversely, high abundance of CLO1 and CLO2 might result in high VLCFAS in TAG in OBs (Miklaszewska et al., 2023). *Ta*CLO1 protein was in fact one of the most abundant proteins detected in OB enriched fractions from Y and M stages in our proteomic analysis in Pennycress (**Figure 7**). Furthermore, mutations in OBAP1 also showed that fatty acids in TAG resulted in increased 18:2 levels and decreases in 20:1 ones in Arabidopsis (López-Ribera et al., 2014). In conclusion, all these results suggest that each type of OB associated proteins has a specific role either in formation (*Ta*SEIPIN1), or stabilization and accumulation (*Ta*OLE5 at the beginning, rest of *Ta*OLE proteins later, together with *Ta*OBAPs and *Ta*CLOs), and that the different ratios and interactions between the different OB-associated proteins may define the OB size, seed oil content and fatty acid composition of the recruited TAG in OBs in Pennycress. Further studies focused on the analysis of such interactions will help to clarify this point.

## Data Availability

The names of the repository/repositories and accession number(s) of the RNA-Seq data can be found at NCBI GEO (GSE256460) or https://doi.org/10.20350/digitalCSIC/16109.

## Supplementary material

Supplementary Figure 1 Motif sequence analysis of Pennycress oleosins with respect to *Brassica napus*.

Supplementary Figure 2
**(A)** Tandem configuration of *OLET* genes (*T1-T7*) in the Pennycress chromosome 6. (B) Expression analysis (FPKM values) of some Pennycress *OLET* genes during Pennycress seed maturation.

Supplementary Figure 3 Motif sequence analysis of Pennycress SEIPINS with respect to *Arabidopsis thaliana* (A) and *Brassica napus* (B).

Supplementary Figure 4 Motif sequence analysis of Pennycress OBAPs with respect to *Arabidopsis thaliana* (A) and *Brassica napus* (B).

Supplementary Table 1 List of primers used for qPCR analysis in this study

Supplementary Table 2 List of different nomenclature of *Arabidopsis thaliana* seed oleosins used in previous papers.

Supplementary Table 3 Main caleosins and stereolisins in *Thlaspi arvense* and their sequence characteristics.

Supplementary Table 4 List of proteins detected in the proteomic analysis of OB-enriched fractions from YELLOW and MATURE Pennycress seeds.
