# Supplementary figures and images for: Transcriptomic and proteomic analysis of oil body associated protein dynamics in the biofuel feedstock Pennycress (Thlaspi arvense)
Source: Front Plant Sci. 2025 Feb 18;16:1530718. doi: 10.3389/fpls.2025.1530718 (PMC11876164; doi:10.3389/fpls.2025.1530718)

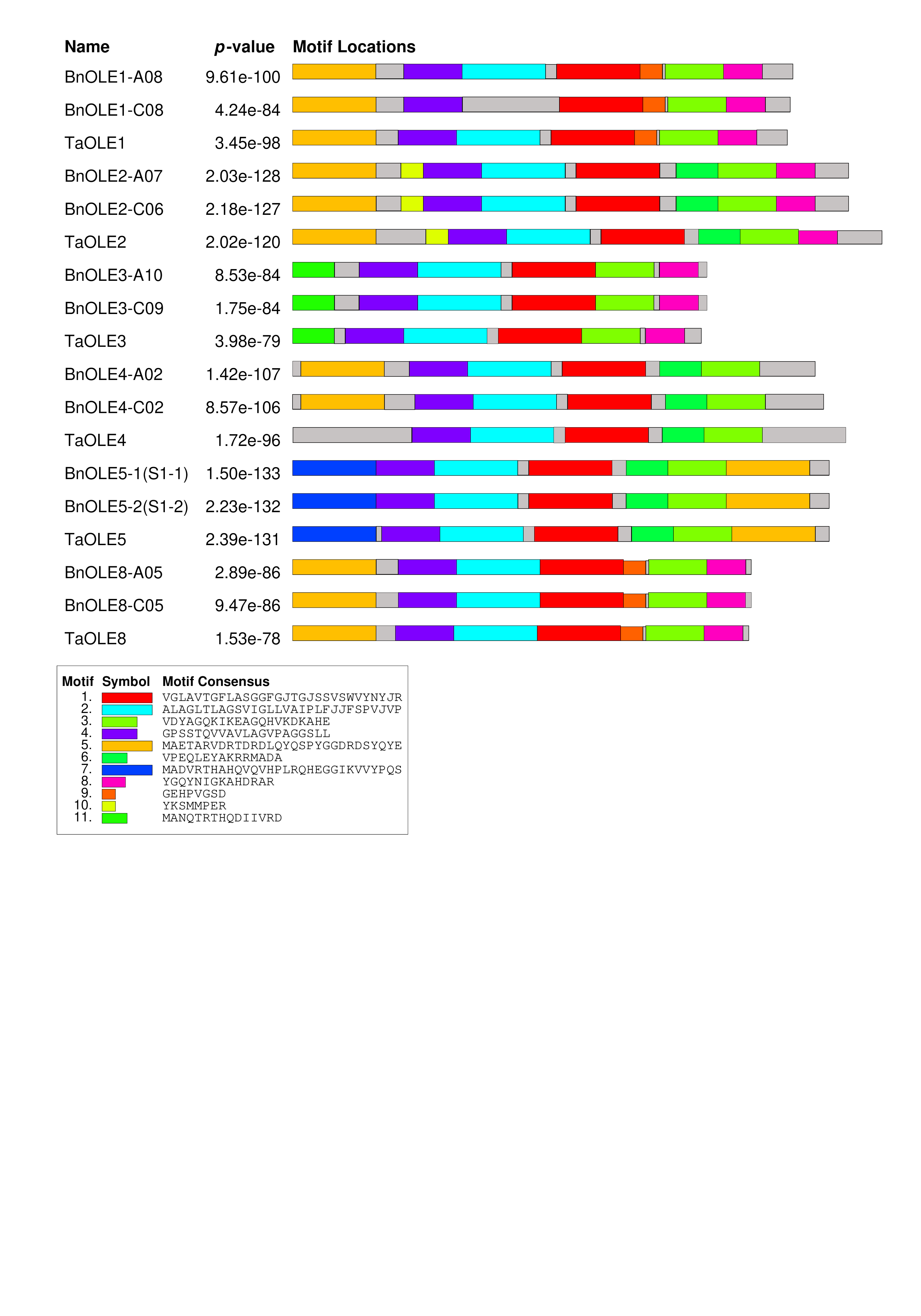

Supplement: Supplementary Figure 1 — Motif sequence analysis of Pennycress oleosins with respect to Brassica napus. [file Image1.tif]

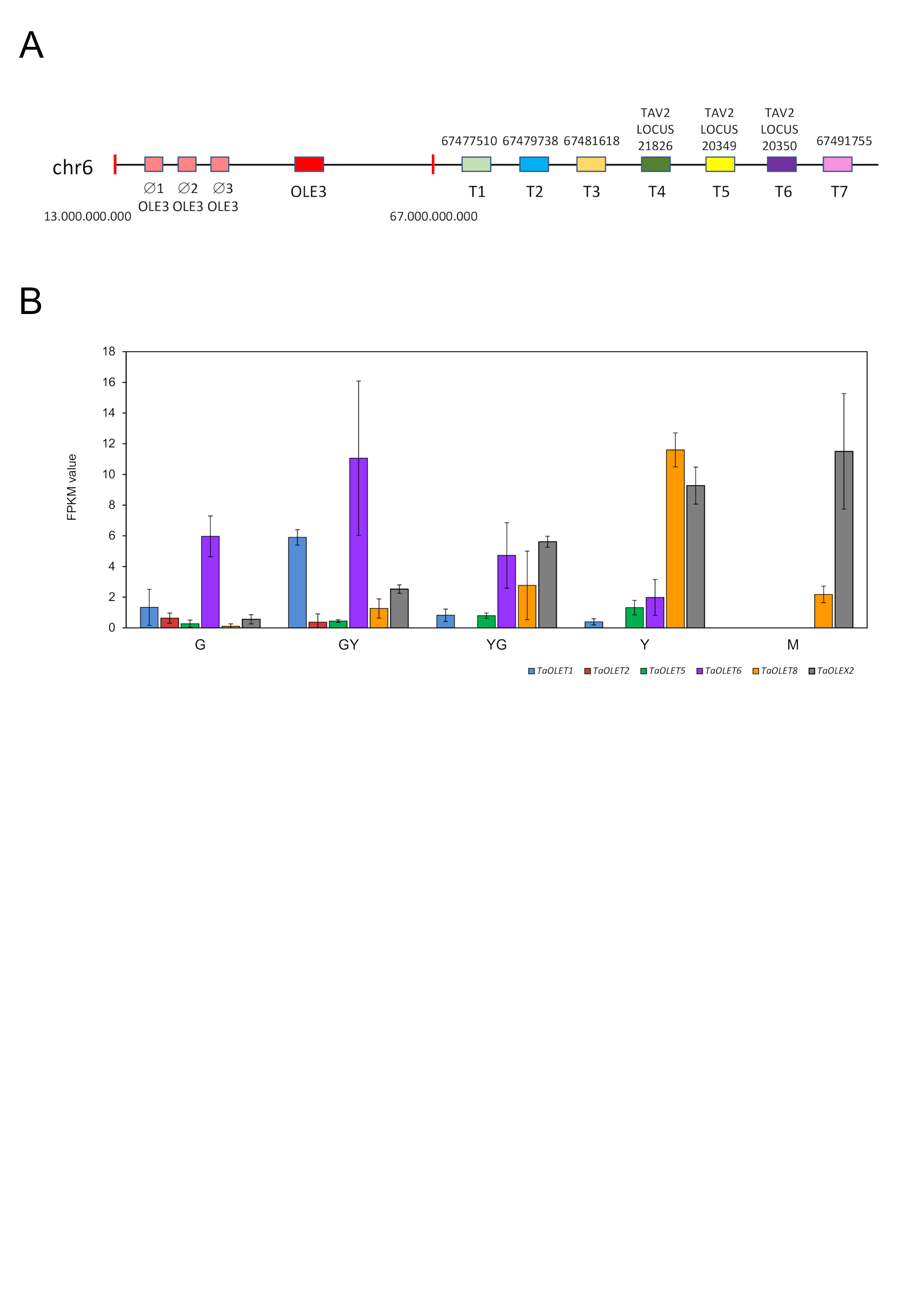

Supplement: Supplementary Figure 2 — (A) Tandem configuration of OLET genes (T1-T7) in the Pennycress chromosome 6. (B) Expression analysis (FPKM values) of some Pennycress OLET genes during Pennycress seed maturation. [file Image2.tif]

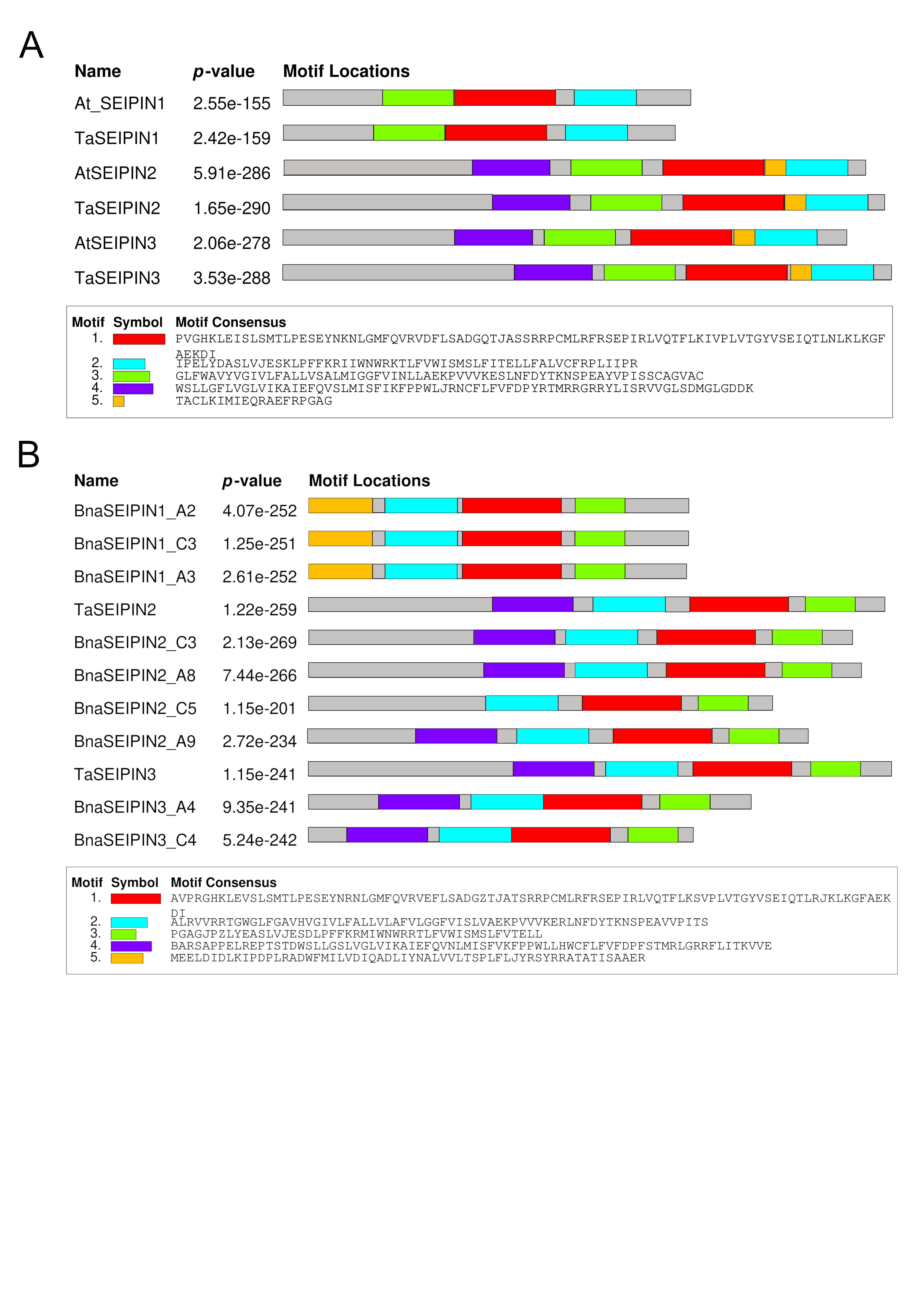

Supplement: Supplementary Figure 3 — Motif sequence analysis of Pennycress SEIPINS with respect to Arabidopsis thaliana (A) and Brassica napus (B). [file Image3.tif]

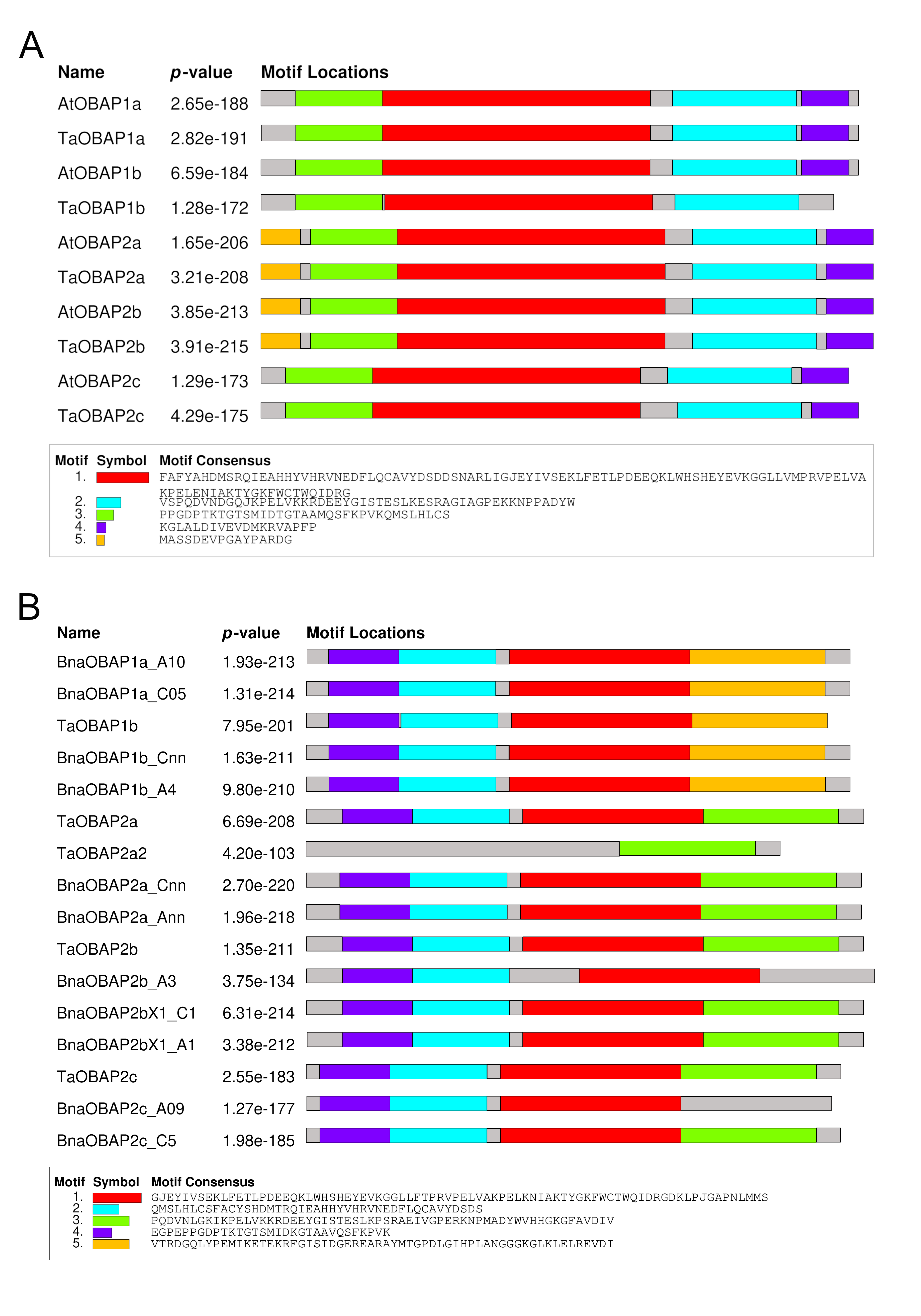

Supplement: Supplementary Figure 4 — Motif sequence analysis of Pennycress OBAPs with respect to Arabidopsis thaliana (A) and Brassica napus (B). [file Image4.tif]
