# Supplementary material for: Transcriptomic and proteomic analysis of oil body associated protein dynamics in the biofuel feedstock Pennycress (Thlaspi arvense)
Source: Front Plant Sci. 2025 Feb 18;16:1530718. doi: 10.3389/fpls.2025.1530718 (PMC11876164; doi:10.3389/fpls.2025.1530718)
Supplement: Supplementary Table 1 — List of primers used for qPCR analysis in this study [file Table1.docx]

| Gene | LOCUS | Primer name | Sequence 5'🡪 3' |  |  |  |
| --- | --- | --- | --- | --- | --- | --- |
| *Ta*OLE1 | TAV2_LOCUS24479 | Ta_qOLE1FW | GAGCACCCACAGGGTTCA |  |  |  |
|  |  | Ta_qOLE1RV | CATGTTCTCCACCAGTATGTTGCT |  |  |  |
| *Ta*OLE2 | TAV2_LOCUS23202 | Ta_qOLE2FW2 | GCACTTGCAATTTCAGTCACC |  |  |  |
|  |  | Ta_OLE2RV | TGGGCCTTCGTAGTATCGT |  |  |  |
| *Ta*OLE3 | TAV2_LOCUS18878 | Ta_qOLE3FW | TGGATCGGATCAGAAGATAGAG |  |  |  |
|  |  | Ta_OLE3RV | TGTTGGTGCTGGATTCCA |  |  |  |
| *Ta*OLE4 | TAV2_LOCUS5704 | Ta_qOLE4FW | AACGGCGTGTGGCTGAC |  |  |  |
|  |  | Ta_OLE4RV | CCACCCTGCTGCTGACCC |  |  |  |
| *Ta*OLE5 | TAV2_LOCUS10168 | Ta_qOLE5FW | CGTTCGTACACATTCACACCA |  |  |  |
|  |  | Ta_qOLE5RV | GGGATAAACCACTTTGATGCCT |  |  |  |
| *Ta*OBAP1a | TAV2_LOCUS320 | Ta_qOBAP1FW | CCTAATATCATGATGGCCGT |  |  |  |
|  |  | Ta_OBAP1aRV | CTCTTGGAACGGACTCGAC |  |  |  |
| *Ta*OBAP1  (*Ta*OBAP1a y *Ta*OBAP1b) | TAV2_LOCUS320 y OU466860.2: 7617497 - 7618636 | Ta_qOBAP1FW | CCTAATATCATGATGGCCG |  |  |  |
|  |  | Ta_qOBAP1 RV | GGGATAAACCACTTTGATGCCT |  |  |  |
| *Ta*OBAP2a | OU466858.1: 9714517 - 9715989 | Ta_qOBAP2abFW | CCCATATTCACGTCTTGTGG |  |  |  |
|  |  | Ta_qOBAP2abRV | CTTCTAGTAGCTCCAAGGGTCC |  |  |  |
| *Ta*OBAP2c | OU466857.2: 4134539 - 4135532 | Ta_qOBAP2c FW | TATGTCTCTGGGTGACAAGTTACC |  |  |  |
|  |  | Ta_qOBAP2cRV | TCAGCTATCTCTGCTCTCTGGTT |  |  |  |
| *Ta*SEIPIN1 | TAV2_LOCUS22247 | Ta_qSEIPIN1 FW | CACTCGTGGTCCTAACCTCAC |  |  |  |
|  |  | Ta_qSEIPIN1RV | TAACCGCTTTTTCCGCTG |  |  |  |
| *Ta*SEIPIN2 | TAV2_LOCUS2470 | Ta_qSEPIN2 FW | AAGAAGAAGAAGACTACGCTGGA |  |  |  |
|  |  | Ta_qSEPIN2 RV | CATCACACCAACTCTTGTCGTT |  |  |  |
