# Supplementary material for: Transcriptomic and proteomic analysis of oil body associated protein dynamics in the biofuel feedstock Pennycress (Thlaspi arvense)
Source: Front Plant Sci. 2025 Feb 18;16:1530718. doi: 10.3389/fpls.2025.1530718 (PMC11876164; doi:10.3389/fpls.2025.1530718)
Supplement: Supplementary Table 2 — List of different nomenclature of Arabidopsis thaliana seed oleosins used in previous papers. [file Table2.docx]

| *Ath Gene* | *UniProtKB accession* | *UniProtKB name* | *TAIR name* | *Name according Siloto et al. (2006)* | *Name according Shimada et al. (2008)* |
| --- | --- | --- | --- | --- | --- |
| AT4G25140 | P29525 | OLE1 | OLE1 | OLEO1 | OLE1 |
| AT5G40420 | Q39165 | OLE2 | OLE2 | OLEO2 | OLE2 |
| AT5G51210 | Q43284 | OLE3 | OLEO3 | OLEO5 | OLE5 |
| AT3G27660 | Q42431 | OLE4 | OLE3, OLEO4 | OLEO4 | OLE3 |
| AT3G01570 | Q9SS98 | OLE5 |  |  | OLE4 |
