# Supplementary material for: Transcriptomic and proteomic analysis of oil body associated protein dynamics in the biofuel feedstock Pennycress (Thlaspi arvense)
Source: Front Plant Sci. 2025 Feb 18;16:1530718. doi: 10.3389/fpls.2025.1530718 (PMC11876164; doi:10.3389/fpls.2025.1530718)
Supplement: Supplementary Table 3 — Main caleosins and stereolisins in Thlaspi arvense and their sequence characteristics. [file Table3.docx]

Tabla S1: Main Caleosins and stereolisins in *T. arvense* genome and their sequence characteristics

| ***Protein*** | ***Ath Gene*** | ***Ta chromosome*** | ***gene position*** | ***gene length*** | ***CDS length*** | ***Ta predicted protein*** | ***% identitty with Ath*** |
| --- | --- | --- | --- | --- | --- | --- | --- |
| CLO1 | AT4G26740 | OU466863.1 | TAV2_LOCUS24828 | 1295 | 738 | CAH2077850.1 | 90.20% |
| CLO2 | AT5G55240 | OU466862.2 | TAV2_LOCUS22044 | 1275 | 732 | CAH2069885.1 | 88.48% |
| CLO3 | AT2G33380 | OU466860.2 | TAV2_LOCUS12356 | 2244 | 723 | CAH2059611.1 | 88.56% |
| SLO1 | AT5G50600/ AT5G50700 | OU466862.2 | TAV2_LOCUS20294 | 1726 | 1050 | CAH2070473.1 | 89.97% |
| SLO2A | AT4G10020 | OU466862.2 | TAV2_LOCUS19638 | 2866 | 1224 | CAH2070610.1 | 90.79% |
| SLO2B |  | OU466857.2 | TAV2_LOCUS2013 | 2206 | 1035 | CAH2035766.1 | 77.26% |

Tabla 2: OB associated proteins in *T. arvense* and *A. thaliana* characteristics and theorical pI.

| ***Protein*** | ***Ta aa*** | ***Ta MW (Da)*** | ***Ta pI*** | ***Ath aa*** | ***Ath MW (Da)*** | ***Ath pI*** |
| --- | --- | --- | --- | --- | --- | --- |
| OLE1 | 177 | 19140.63 | 9.39 | 173 | 18569.09 | 9.43 |
| OLE2 | 211 | 22349.57 | 9.32 | 199 | 21279.37 | 9.36 |
| OLE3 | 146 | 15342.78 | 9.9 | 141 | 14852.2 | 9.86 |
| OLE4 | 198 | 20642.37 | 6.5 | 191 | 20313.15 | 6.91 |
| OLE5 | 192 | 20725.81 | 7.11 | 183 | 19754.7 | 7.11 |
| OLE8 | 164 | 18010.68 | 8.93 | 166 | 18134.78 | 9.34 |
| OBAP1a | 241 | 27248.34 | 6.11 | 241 | 27293.34 | 6.22 |
| OBAP1b | 231 | 25915.45 | 5.49 | 241 | 26759.49 | 5.69 |
| OBAP2a | 247 | 27725.62 | 5.99 | 247 | 27845.93 | 5.92 |
| OBAP2b | 247 | 27773.99 | 7.04 | 247 | 27747.95 | 7.64 |
| OBAP2c | 241 | 27429.13 | 5.66 | 237 | 26875.58 | 5.98 |
| SEIPIN1 | 354 | 40852.23 | 10 | 368 | 42479.03 | 10.22 |
| SEIPIN2 | 543 | 61146.27 | 5.21 | 526 | 59677.23 | 5.05 |
| SEIPIN3 | 549 | 61504.97 | 5.37 | 509 | 57415.51 | 5 |
| LDAP1 | 238 | 25782.41 | 8.39 | 240 | 26425.14 | 8.9 |
| LDAP2 | 249 | 28079.44 | 4.32 | 235 | 26642.18 | 4.42 |
| LDAP3 | 239 | 26545.71 | 8.82 | 246 | 27194.48 | 8.31 |
| CLO1 | 245 | 27958.92 | 5.75 | 245 | 28038.04 | 5.81 |
| CLO2 | 243 | 27916.71 | 5.6 | 243 | 27875.58 | 5.62 |
| CLO3 | 240 | 27258.81 | 5.32 | 236 | 26600.04 | 5.17 |
| SLO1 | 349 | 39083.96 | 6.34 | 349 | 39086.93 | 5.91 |
| SLO2A | 407 | 45277.8 | 6.77 | 389 | 43441.75 | 6.41 |
| SLO2B | 344 | 39143.97 | 5.86 |  |  |  |
